# Two deeply conserved non-coding sequences control *PLETHORA1/2* expression and coordinate embryo and root development

**DOI:** 10.1016/j.xplc.2025.101466

**Published:** 2025-07-29

**Authors:** Merijn Kerstens, Yvet Boele, Abraham Morales-Cruz, Chris Roelofsen, Peng Wang, Leo A. Baumgart, Ronan O'Malley, Gabino Sanchez-Perez, Ben Scheres, Viola Willemsen

**Affiliations:** 1 Cluster of Plant Developmental Biology, Cell and Developmental Biology, Wageningen University & Research, Droevendaalsesteeg 1, 6708 PB Wageningen, the Netherlands; 2 Department of Terrestrial Ecology, Netherlands Institute of Ecology (NIOO-KNAW), Droevendaalsesteeg 10, 6708 PB Wageningen, the Netherlands; 3 US Department of Energy, Joint Genome Institute, Lawrence Berkeley National Laboratory, Berkeley, CA 94720, USA; 4 Department of Human Genetics, University of Chicago, Chicago, IL 60637, USA; 5 Department of Molecular Genetics, Utrecht University, Padualaan 8, 3584 CH Utrecht, the Netherlands

**Keywords:** PLETHORA, conserved non-coding sequence, angiosperm, transcriptional regulation, root meristem, embryogenesis

## Abstract

Conserved non-coding sequences (CNSs) are integral elements of transcriptional regulation. Transcriptional tuning of *PLETHORA* (*PLT*) genes that encode master regulators of plant development is vital for embryogenesis and meristematic function. However, how the expression of *PLT* genes is modulated through CNSs remains unclear. Through motif-based mining of upstream sequences in 120 angiosperm genomes, we identified 21 conserved and lineage-specific CNSs, two of which are unusually long, similar, and colinear within eudicots. Using *Arabidopsis thaliana*, we demonstrate that these two deeply conserved elements, which we named BOX1 and BOX2, control *PLT1* and *PLT2* expression. CRISPR mutants within these elements specifically reduced *PLT* expression levels, and reporter lines revealed that deletion of either or both BOXes altered and/or abrogated the *PLT2* expression pattern in the root tip, affecting the ability to rescue the *plt1 plt2* double mutant. We further show that the influence of these elements on expression patterns is already exerted during embryogenesis and functional in the context of the early embryo. Finally, we reveal the existence of a BOX-mediated autoregulatory feedback loop that, in large part, explains CNS influence on expression patterns. We thus uncover a transcriptional mechanism by which genes encoding master regulators of embryo and root meristem development are regulated.

## Teaser

This study reports the identification of two colinear, deeply conserved non-coding sequences upstream of eudicot *PLETHORA1/2* orthologs that are essential for embryogenesis and root development in *Arabidopsis thaliana*. These sequences are bound by PLETHORAs and thus function in an autoregulatory feedback loop.

## Introduction

Regulation of gene expression lies at the heart of cellular function in all organisms. It is a complex quantitative process mediated by the initial recruitment of transcriptional machinery to the transcription start site and binding of transcription factors (TFs) to *cis*-regulatory sequences in the dynamic context of chromatin accessibility (Andersson and Sandelin, 2020; Kim and Wysocka, 2023). *cis-*regulatory sequences are stretches of non-coding DNA under purifying selection, implying that they are functionally conserved across species despite not serving as templates for transcription themselves. In mammals, such conserved non-coding sequences (CNSs) are generally long (>100 bp), strongly conserved (>70% identity), and located at distances spanning up to multiple millions of base pairs from their neighboring genes (Nobrega et al., 2003; Bejerano et al., 2004; Sandelin et al., 2004; Woolfe et al., 2005). Compared with their mammalian counterparts, angiosperm CNSs are much shorter, less conserved, and closer to their associated genes (Thomas et al., 2007; Baxter et al., 2012; Haudry et al., 2013; Turco et al., 2013; Burgess and Freeling, 2014; de Velde et al., 2014; Hettiarachchi et al., 2014; Van de Velde et al., 2016; Lai et al., 2017), despite both lineages sharing a similar evolutionary divergence time (Bininda-Emonds et al., 2007; Magallón et al., 2015). Similar to those of vertebrates, angiosperm CNSs are enriched in sites of accessible chromatin and TF-binding sites and often occur near developmental regulators (Burgess and Freeling, 2014; de Velde et al., 2014). A distinct group of mammalian CNSs exist in the form of ultraconserved non-coding elements, which were initially defined as identical ≥200-bp sequences shared among the human, mouse, and rat genomes (Bejerano et al., 2004). Recently, an updated definition of ≥100-bp sequences with ≥97% sequence identity in ≥50% of placental mammalian orders was proposed (Cummins et al., 2024). Although CNSs do not occur at such high conservation levels across angiosperms, highly conserved sequences reminiscent of ultraconserved non-coding elements have been described (Kritsas et al., 2012; Haudry et al., 2013). For instance, Kritsas et al. (2012) defined ultraconserved-like non-coding elements as >55 bp with ≥85% identity between *Arabidopsis* (*Arabidopsis thaliana*) and grape (*Vitis vinifera*; divergence ∼115 million years ago) and between *Brachypodium distachyon* and rice (*Oryza sativa*; divergence ∼50 million years ago).

PLETHORAs (PLTs) are plant-specific TFs from the euAINTEGUMENTA branch of the APETALA2/ETHYLENE-RESPONSIVE ELEMENT BINDING PROTEIN superfamily and are master regulators of key developmental processes, including embryogenesis (Smith and Long, 2010; Chen et al., 2022; Kerstens et al., 2024) and root apical meristem (RAM) maintenance (Aida et al., 2004; Galinha et al., 2007). In angiosperms, PLTs can be divided into four clades (i.e., PLT1/2, PLT3/7, BBM (BABY BOOM)/PLT4, and PLT5) based on sequence similarity, with each clade exhibiting various levels of lineage-specific copy number variation and/or levels of synteny (Kerstens et al., 2020). Several lines of evidence demonstrate that PLTs are deeply conserved and act redundantly across tissues and developmental phases. In *Arabidopsis*, expression of any *PLT* from the *PLT2* promoter in the RAM can rescue the *plt1 plt2* phenotype, in which the RAM stem-cell niche differentiates over the course of 6–8 days (Galinha et al., 2007; Santuari et al., 2016). In addition, ectopic expression of *PLT1* or *PLT3* in the zygote using *pPLT2* or *pBBM* is sufficient to allow progression of early embryogenesis in *plt2 bbm* (Kerstens et al., 2024). Moreover, all six PLT TFs bind an AINTEGUMENTA-like (ANT-like) consensus motif and collectively activate a set of target genes that induce meristematic potential in each RAM cell lineage, the zygote, and the shoot apical meristem (O’Malley et al., 2016; Santuari et al., 2016; Kerstens et al., 2024).

If PLT function is conserved from the zygote to post-embryonic meristems in *Arabidopsis*, then PLT localization rather than PLT identity must be crucial for robust development in land plants. However, the demarcation of *PLT* expression domains through transcriptional regulation is poorly understood. In this study, we systematically mined the promoters of all PLT clades across >100 angiosperm species to identify CNSs that could potentially regulate *PLT* expression. We discovered two CNSs in the *PLT1/2* promoter of eudicots and experimentally verified their contribution to the establishment of *AtPLT2* gene expression patterns in different organs as well as the effect of deletions on RAM maintenance and embryo development. Finally, we reveal that these CNSs contain an autoregulatory PLT-binding site that is required for their role in shaping *PLT2* expression patterns.

## Results

### *PLT* promoters contain conserved and lineage-specific motifs

**Figure 1:**
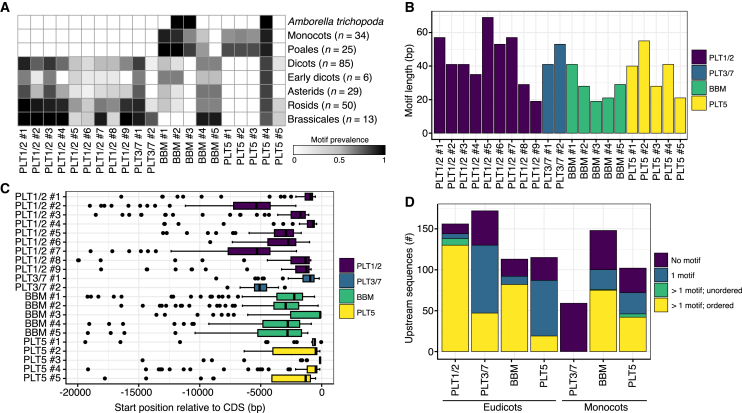
*PLT* homologs are characterized by distinct upstream conserved motifs. **(A)** Grayscale heatmap displaying the prevalence of identified PLT motifs (columns) across angiosperm lineages (rows). **(B and C) (B)** Motif lengths in each PLT homolog clade and **(C)** their positions relative to the start codon. **(D)** Occurrence of colinear and unordered promoter motifs in each PLT clade.

Given the demonstrated key importance of the regulation of *PLT* expression, we searched for CNSs *de novo* by analyzing the 20 kb directly upstream of the annotated coding sequences (CDSs) of all *PLT* homologs in a selection of PLAZA5.0 monocot and eudicot species panels, comprising 34 monocot and 85 eudicot species, *Amborella trichopoda* (sister species to angiosperms), and 9 distant non-angiosperm species (Van Bel et al., 2022). We found 817 PLT homologs across four clades, i.e., 156 PLT1/2, 218 PLT3/7, 241 BBM, and 202 PLT5 (Supplemental Figure 1A and 1B; Supplemental Table 1); only the last three clades occurred in all angiosperms, and PLT1/2 occurred only in eudicots, in line with previous work (Kerstens et al., 2020; Rodríguez Herrera et al., 2025). We then used MEME (Bailey et al., 2015) to search for conserved elements (15–100 bp) in upstream sequences of each *PLT* clade (20 kb or until the upstream gene) separately for monocots and eudicots. After removal of repetitive motifs and those with low conservation, 21 of 64 significant motifs represented putative CNSs across *PLT* upstream sequences (Supplemental Table 2). We then re-searched for these motifs across all upstream sequences (Figure 1A and Supplemental Figure 2; Supplemental Table 3). Motifs were generally unique to the *PLT* clade in which they were originally discovered, with the exception of PLT5 #4, which was also present upstream of a subset of eudicot *PLT3/7* genes (Supplemental Figure 3). Nine motifs were present upstream of *PLT1/2* homologs, with varying degrees of lineage specificity (Figure 1A and Supplemental Figure 2). Two upstream motifs were identified in the *PLT3/7* clade in eudicot species, one of which was Brassicales specific, whereas no PLT3/7 motifs were identified in monocots (Figure 1A and Supplemental Figure 2). Five motifs were found upstream of *BBM* homologs; one was monocot specific, one was eudicot specific, and three were shared between monocots and eudicots but lost in Brassicales (Figure 1A and Supplemental Figure 2). For PLT5, we found one motif consistently shared between monocots and eudicots, three monocot/Poales-specific motifs, and one rosid-specific motif (Figure 1A and Supplemental Figure 2). PLT motifs varied in length, ranging from 19 to 69 bp, with the largest motifs found upstream of *PLT1/2* homologs (Figure 1B; Supplemental Table 2). Motifs showed various degrees of location specificity but were generally positioned within 5 kb of the CDS (Figure 1C; Supplemental Table 3). Strikingly, in cases where multiple motifs were present within an upstream region, their relative positional order was highly conserved (Figure 1D). Thus, upstream sequences of PLTs contain CNSs that are conserved by sequence, position, and order across angiosperms.

### Eudicot *PLT1/2* promoters harbor two deeply conserved non-coding sequences

**Figure 2:**
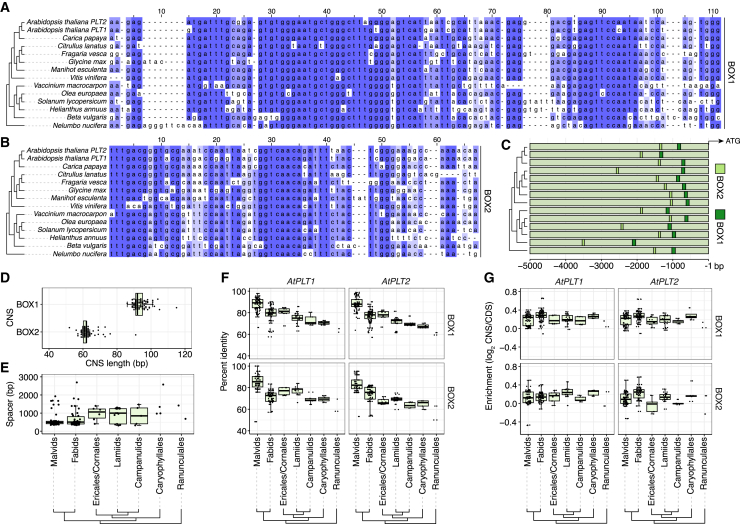
Two deeply conserved non-coding sequences are upstream of *PLT1/2* orthologs. **(A and B)** Multiple sequence alignment of **(A)** BOX1 and **(B)** BOX2 across 14 representative eudicot species. Sequence conservation levels scale with the intensity of blue shading. The phylogeny represents species divergence. **(C)** Positions of the CNSs relative to the start of the annotated CDS. Dendrogram and species order as in **(A) and (B)**. **(D)** CNS length per ortholog. **(E)** Spacer distance between BOX1 and BOX2 in sequences of both CNSs across major phylogenetic taxa. Not shown are three outliers for *Pisum sativum* (17 906 bp), *Gossypium hirsutum* (15 798 bp), and *Gossypium raimondii* (15 228 bp). **(F)** Percentage identity match of *Arabidopsis PLT1* and *PLT2* CNSs with other BOX sequences across eudicot lineages. **(G)** Log_2_ enrichment of *Arabidopsis* BOX percentage identity over percentage identity matches with the corresponding ortholog CDS. Boxplots were drawn when *n* > 4. *Arabidopsis PLT1* and *PLT2* sequences were excluded from malvids in **(F) and (G)**.

Within the upstream sequences of *PLT1/2* orthologs, the PLT1/2 #1 and PLT1/2 #3 motifs seemed particularly well conserved and were present in almost all eudicot species (Figure 1A). To assess the conservation levels of these two CNSs in more detail, we made multiple sequence alignments of the motif regions and 100-bp flanking sequences. We found that the most upstream motif (PLT1/2 #3) was embedded in a highly conserved CNS of, on average, ∼60 bp in 128 of 156 orthologs (82%), whereas the motif closer to the CDS (PLT1/2 #1) was positioned in an even longer CNS of ∼90 bp in 140/156 orthologs (90%) (Figure 2A–2D and Supplemental Figure 4; Supplemental Table 4). We named the CNSs BOX2 and BOX1, respectively. BOX1 and BOX2 co-occurred upstream of 127/156 orthologs (81%)—always in the same order and primarily within the first 2.5 kb of the CDS start site (Figure 2C and Supplemental Figure 4). Moreover, BOX2 occurred at least once within the analyzed 20 kb in 73/80 species with *PLT1/2* orthologs (91%) and BOX1 in 77/80 species (96%), and co-occurrence was observed at least once in 72/80 species (90%). Upstream of a handful of orthologs, BOX2 (*n* = 5) and BOX1 (*n* = 3) were identified twice (Supplemental Figure 4). The CNSs were separated by a spacer region that varied, on average, from ∼500 bp in rosids (malvids and fabids) to ∼1000 bp in asterids (Ericales/Cornales, lamiids, campanulids, and Caryophyllales), although longer spacers were observed in individual species (Figure 2E).

Because our multiple sequence alignments suggested deep conservation and we aimed to quantify the degree of similarity, we extracted the percentage identity scores for each pairwise CNS comparison from the alignments. Within the analyzed species panel, BOX1 was slightly more conserved than BOX2, and the most frequent percentage identity levels were between 60% and 90%, with related species sharing more sequence similarity than more distant relatives (Supplemental Figure 5A and 5B). For instance, *Arabidopsis* BOX1 and BOX2 shared roughly 90% and 85% identity with malvid BOX sequences, ∼80% and ∼75% with fabid sequences, and ∼70% with Caryophyllales sequences (Figure 2F). We then wondered how these conservation levels related to those of the *PLT1/2* protein CDSs. Strikingly, pairwise BOX percentage identity was higher, on average, than that of the corresponding full-length CDS across all eudicot lineages, demonstrating an exceptional degree of conservation over ∼130 million years of eudicot evolution (Hertweck et al., 2015) (Figure 2G and Supplemental Figure 5C and 5D). The high conservation level, large size, and strict occurrence order of BOX1 and BOX2 are reminiscent of those of ultraconserved non-coding sequences in placental mammals.

### *Arabidopsis PLT1/2* CNSs regulate expression and shape root development

**Figure 3:**
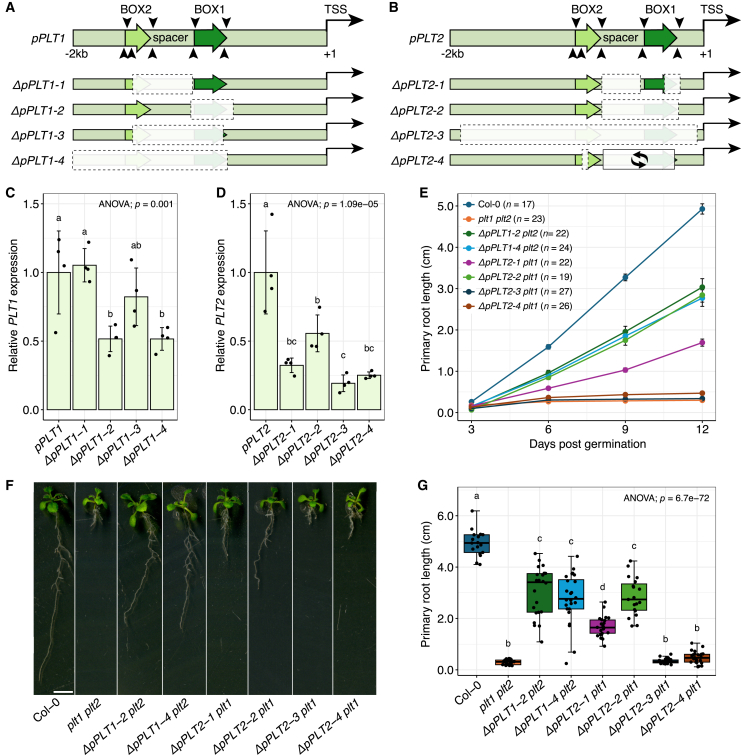
*Arabidopsis PLT1* and *PLT2* expression is controlled by upstream CNSs. **(A and B)** Promoter models and CRISPR alleles of the BOX regions in **(A)***pPLT1* and **(B)***pPLT2*. Arrowheads indicate sgRNA sites, dashed boxes represent deletions, and the closed box with circular arrows denotes an inversion. Indels of 1 bp are not indicated. **(C and D)** Relative expression (±SD) of **(C)***PLT1* and **(D)***PLT2* in the *plt2-2* and Col-0 backgrounds, respectively, from wild-type and mutant promoter alleles. Wild-type promoter expression is set to 1. Each bar is based on four biological replicates. Significance was determined by Tukey’s HSD *post hoc* tests. **(E)** Time course of primary root growth (±SEM) among wild-type, *plt1 plt2-cr*, and *ΔpPLT* alleles in the *plt1-cr* or *plt2-cr* background. **(F)** Representative images of 12-dpg seedlings. Scale bar, 5 mm. **(G)** Primary root length at 12 dpg with Tukey’s HSD *post hoc* tests. Data are derived from **(E)**.

If BOX1 and BOX2 are CNSs under strong selection, then they should be functional elements that direct the expression of *PLT1/2* orthologs. To assess this notion, we used CRISPR-Cas9 to mutate the BOX regions upstream of *Arabidopsis PLT1* and *PLT2*, which likely arose from an ancestral *PLT1/2* gene through a Brassicaceae-specific whole-genome duplication event (Kerstens et al., 2020). We generated four deletion alleles per promoter, ranging from partial deletions to complete loss of BOX1 and BOX2 to a sequence inversion (Figure 3A and 3B; Supplemental Table 5). Expression of the alleles was then quantified in root tips by RT–qPCR. In *pPLT1*, loss of BOX1 (*ΔpPLT1-2*) reduced expression roughly two-fold in comparison to the full *PLT1* promoter, and so did loss of the entire CNS region, including the ∼500 bp upstream of BOX2 (*ΔpPLT1-4*; Figure 3C). A deletion of the spacer and two-thirds of BOX2 (*ΔpPLT1-1*), as well as a deletion of the spacer, two-thirds of BOX2, and all but 10 bp of BOX1 (*ΔpPLT1-3*), had no clear effect (Figure 3C). In *pPLT2*, all four alleles exhibited reduced *PLT2* expression, including an allele in which 18 bp were deleted from BOX2 together with an inverted BOX1 spacer module (*ΔpPLT2-4*; Figure 3D), indicating that BOX1 and BOX2 control *pPLT1/2* expression.

We next asked whether the observed reductions in expression affected primary root development. To this end, *ΔpPLT1* and *ΔpPLT2* alleles with significantly reduced expression were crossed in the *plt2-cr* and *plt1-cr* background, respectively. In this way, primary root length served as a proxy for promoter function, with mutants harboring inactivated promoters in the *plt1-cr* or *plt2-cr* single-mutant background resembling the *plt1 plt2* double mutant. In contrast to Columbia-0 (Col-0), which exhibits the same primary root growth as single *plt-cr* mutants (Supplemental Figure 6), *ΔpPLT2-3 plt1-cr* and *ΔpPLT2-4 plt1-cr* roots showed rapid growth arrest after 6 days post germination (dpg) and phenocopied the *plt1 plt2-cr* mutant (Figure 3E–3G). The primary root in other *ΔpPLT2 plt1-cr* and *ΔpPLT1 plt2-cr* lines did not show growth arrest but grew more slowly than that of Col-0 (Figure 3E). At 12 dpg, *ΔpPLT1-2 plt2-cr*, *ΔpPLT1-4 plt2-cr*, and *ΔpPLT2-1 plt1-cr* roots were ∼40% shorter than those of Col-0, and *ΔpPLT2-2 plt1-cr* roots were ∼60% shorter (Figure 3F and 3G). Deletion and inversion in the BOX regions of *pPLT1/2* thus perturb root growth in *Arabidopsis*.

### BOX1 and BOX2 synergistically control *PLT2* expression in a cell-type-specific manner

**Figure 4:**
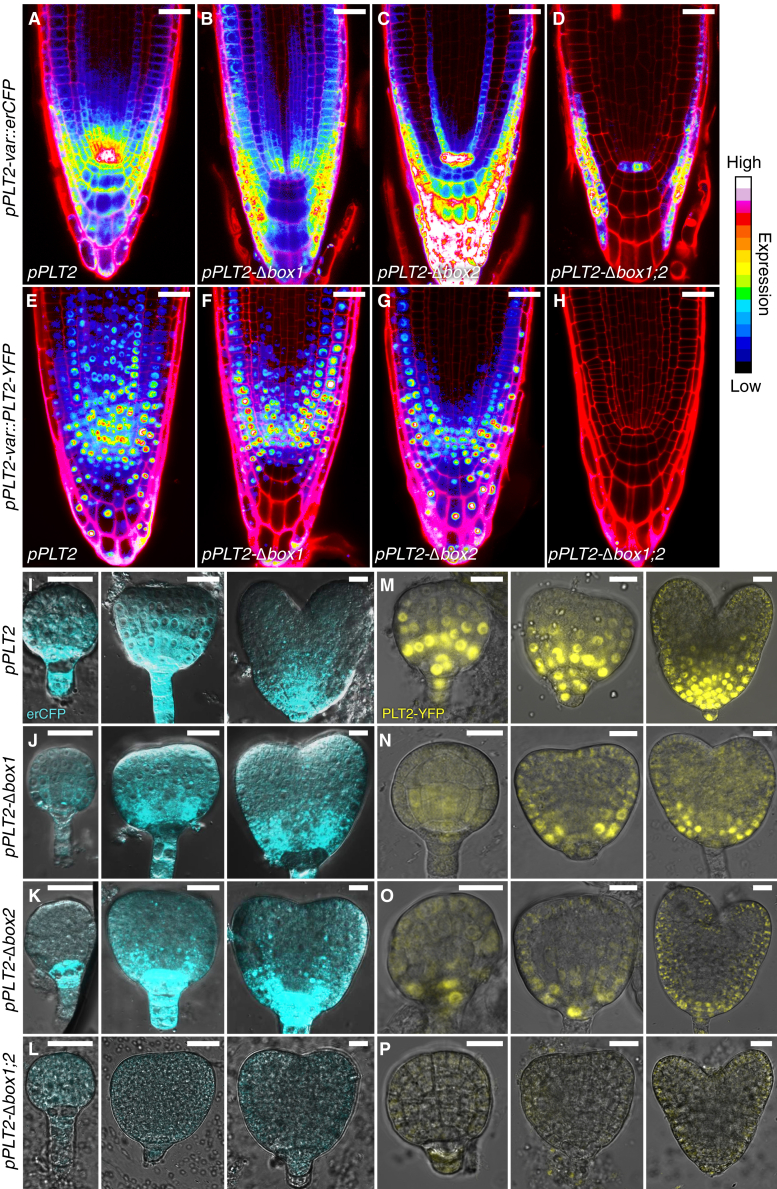
Deletions of BOX1 and BOX2 perturb *PLT2* expression domains. **(A–D)** Expression of **(A)***pPLT2::erCFP* and **(B–D)** variants (*pPLT2-var*) in 5-dpg Col-0 root tips stained with propidium iodide (PI). **(E–H)** PLT2-YFP driven from **(E)***pPLT2* and **(F–H)** variants in 4-dpg Col-0 PI-stained root tips. erCFP and PLT2-YFP signals are shown according to the “16 color LUT.” **(I–P)** Expression of **(I)***pPLT2::erCFP* and **(J–L)** variants in globular, triangular, and heart-stage embryos. Scale bars, 15 μm. PLT2-YFP driven from **(M)***pPLT2* and **(N–P)** variants in globular, triangular, and heart-stage Col-0 embryos.

Our CRISPR alleles harbored coarse promoter alterations that did not allow us to distinguish BOX-specific functions. To dissect the roles of these elements in greater detail, we chose *Arabidopsis PLT2* over *PLT1* because it is expressed more broadly in the RAM (Galinha et al., 2007), has an additional role during early embryogenesis (Kerstens et al., 2024), and showed stronger phenotypes in our CRISPR assay (Figure 3C–3G). We first studied the expression patterns of wild-type *pPLT2* (5.8 kb; a length that functionally complements *plt1 plt2* [Galinha et al., 2007] and *plt2 bbm* [Kerstens et al., 2024] phenotypes) and promoter variants in which one BOX or both elements were deleted, driving *erCFP* in root tips. Wild-type *pPLT2* expressed *erCFP* in a graded manner in all RAM cell types, with the highest expression peak in the stem cell niche, including in the entire columella and early vascular cells (Figure 4A). By contrast, *erCFP* expression was reduced in the quiescent center (QC) and columella when driven by *pPLT2-Δbox1*, whereas *pPLT2-Δbox2* caused high expression in the QC, columella, and lateral root cap and loss of expression in the cortex and vascular bundle (Figure 4B and 4C). Deletion of both BOXes (*pPLT2-Δbox1;2*) severely disrupted expression, with signal remaining in the lateral root cap and weakly in the QC and endodermis (Figure 4D). To verify that the observed expression patterns were not artifacts from promoter reporter lines, we fused *YFP* to genomic *PLT2* and expressed the translational fusion protein from the same promoter variants. We observed expression patterns similar to those in the transcriptional fusion lines (Figure 4E–4H). Specifically, we again noticed prominent expression gaps in the columella and vasculature caused by deletion of BOX1 and BOX2, respectively, and we could not detect a signal when *PLT2-YFP* was driven from *pPLT2-Δbox1;2*. We also observed a shorter basipetal gradient and wild-type expression in the QC of seedlings harboring the *pPLT2-Δbox1* translational fusion (Figure 4F). Jointly, these findings reveal specific roles for BOX1 and BOX2 in the spatiotemporal regulation of *pPLT2* expression.

To exclude the possibility that BOX deletions affect protein distribution through *cis* interactions with promoter elements farther upstream and in the spacer region, we expressed *PLT2-YFP* from truncated promoters containing either both BOXes and the spacer (*pPLT2-1.7kb*) or only BOX1 (*pPLT2-1.3kb* [Galinha et al., 2007]). Whereas removal of 4.1 kb upstream of the BOX regions did not change PLT2-YFP localization compared with the full promoter, *pPLT2-1.3kb* failed to generate signal in the vasculature, thereby phenocopying *pPLT2-Δbox2* (Supplemental Figure 7A–7C). This shows that the altered expression patterns result specifically from loss of the BOX sequences.

Because BOX1 and BOX2 were exclusively found in the same order in the promoters of *PLT1/2* orthologs (Supplemental Figure 4), we also expressed *PLT2-YFP* from a promoter variant in which the BOX1 and BOX2 sequences were swapped without changing their orientation (*pPLT2-swap*). Swapping the CNSs caused the formation of a shorter upward gradient in the vasculature, while columella expression was retained (Supplemental Figure 7D). These data suggest that the order of the BOX regions is required only for specific aspects of *pPLT2* expression. Taken together, our results demonstrate that BOX1 and BOX2 individually and synergistically define *pPLT2* activity independently of their order and upstream sequence.

### BOX-deletion defects are initiated during embryogenesis

*PLT2* expression is initiated in the zygote and maintained throughout embryogenesis, becoming restricted over time to the lower tier of the embryo proper and the hypophyseal cell, then converging on the progenitor cells constituting the future RAM in a gradient (Kerstens et al., 2024). Because the RAM is already established during embryogenesis (Scheres et al., 1994), we wondered whether aberrant *PLT2* expression resulting from BOX deletion already occurred during embryogenesis. Using our transcriptional *erCFP* reporter lines, we observed that loss of BOX1 led to weaker expression in both the suspensor and the lower tier of the embryo proper at the globular stage, later manifesting as low QC progenitor expression and loss of columella progenitor expression at the heart stage (Figure 4I and 4J). Deletion of BOX2 increased *pPLT2* activity in the QC and columella progenitor tissues at the globular and heart stage but abolished activity in the vascular tissue (Figure 4K), and absence of both CNSs abrogated embryonic expression entirely (Figure 4L). These expression patterns are in strong congruence with those observed in the root tip. Our translational reporter lines further corroborated the parallels between RAM and late-embryo *PLT2* expression, including the two truncated promoter variants that mimicked the BOX deletion lines (Figure 4M–4P and Supplemental Figure 8). Thus, the erratic transcriptional and translational expression patterns in roots due to loss of BOX1 and/or BOX2 are already manifested during embryogenesis.

### Aberrant *PLT2* expression compromises developmental functions

**Figure 5:**
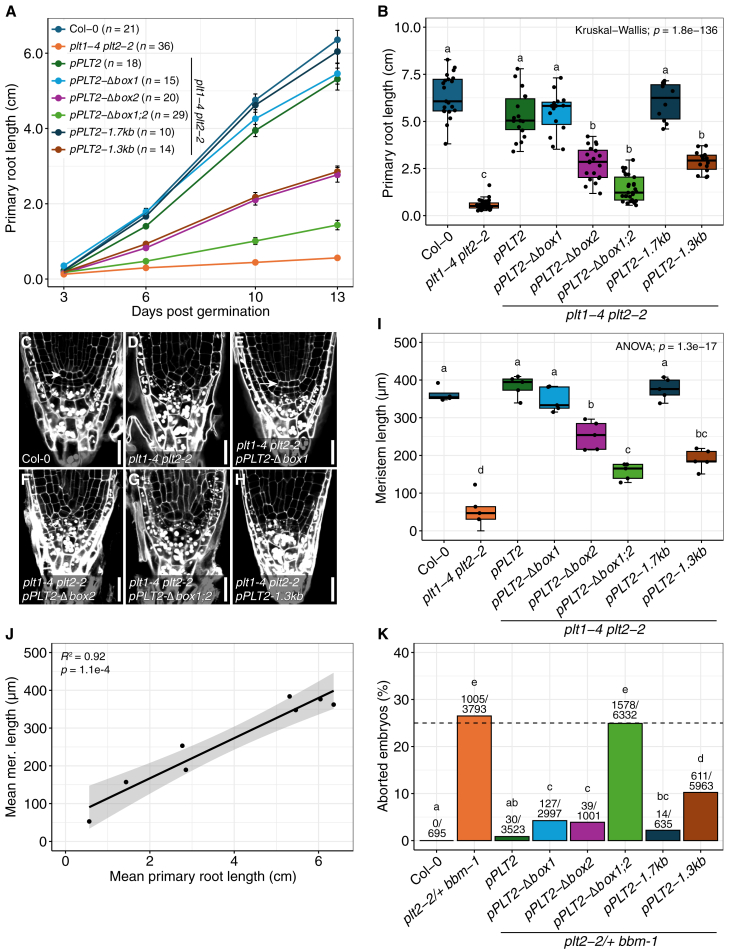
BOX1 and BOX2 are functional developmental modules. **(A)** Time course of primary root growth (±SEM) among Col-0, *plt1-4 plt2-2*, and *plt1-4 plt2-2* mutants complemented with *PLT2-YFP* expressed from promoter variants. **(B)** Primary root length at 13 dpg with Dunn’s *post hoc* tests with Benjamini–Hochberg correction. Data are derived from **(A)**. **(C–H)** Schiff staining of RAMs at 5 dpg. Arrows indicate the QC. Scale bars, 20 μm. **(I)** Meristem length at 7 dpg in Col-0 (*n* = 4) and the (complemented) *plt1-4 plt2-2* lines (*n* = 5) with Tukey’s HSD *post hoc* tests. **(J)** Linear correlation between the mean primary root lengths (PRLs) at 13 dpg **(B)** and mean meristem lengths at 7 dpg **(I)** of the eight analyzed genotypes. The shaded area denotes the 95% confidence interval. **(K)** Percentage of embryo-lethal offspring in selfings of Col-0, *plt2-2/+ bbm-1*, and *plt2-2/+ bbm-1* complemented with translational fusions. The dashed line at 25% shows the expected embryo lethality level according to Mendelian segregation of the *plt2-2 bbm-1* genotype. Numbers indicate aborted and total embryos. Statistical groups were derived from pairwise two-proportion *z*-tests with Yates’ continuity and Bonferroni correction.

To understand what the altered PLT2 domains mean in a developmental context, we introduced the described translational reporter lines into *plt1-4 plt2-2* and quantified root growth over time to assess their ability to complement the short root phenotype of this mutant. Whereas the *pPLT2*, *pPLT2-1.7kb*, *pPLT2-Δbox1*, and *pPLT2-swap* promoters were each able to fully restore the developmental defects of *plt1-4 plt2-2*, *pPLT2-Δbox2*, *pPLT2-1.3kb*, and *pPLT2-Δbox1;2* could only partially complement the phenotype, the last to only a small degree (Figure 5A and 5B and Supplemental Figure 9A and 9B). Upon microscopy analysis of the BOX-deletion root tips at 5 dpg, the clear delineation of the QC and underlying columella stem cells was lost in the *pPLT2-Δbox2* and *pPLT2-Δbox1;2* complementation lines, whereas root tips complemented with *pPLT2-Δbox1* were unaffected (Figure 5C–5H). Indeed, root length correlated well with meristem size, with shorter roots having shorter meristems (Figure 5I and 5J). These data indicate that BOX2 is required for meristem maintenance in the RAM and that it can compensate for loss of BOX1, but not vice versa.

Given the altered *PLT2* expression patterns observed during embryogenesis in our BOX-deletion lines, we sought to determine whether these CNSs are also required earlier in this process. To this end, we introduced the same constructs in *plt2-2/+ bbm-1*, whose homozygous progeny is embryonic lethal and arrests shortly after or at the zygote stage (Kerstens et al., 2024). We then quantified embryonic lethality by counting the percentage of aborted seeds in each line. Only the full *PLT2* promoter was able to reduce embryo lethality to the wild-type level, closely followed by *pPLT2-1.7kb* and *pPLT2-swap* (Figure 5K and Supplemental Figure 9C). Driving *PLT2-YFP* from *pPLT2-Δbox1* or *pPLT2-Δbox2* in large part rescued the *plt2-2 bbm-1* phenotype and *pPLT2-1.3kb*, to a lesser degree (Figure 5K). In contrast to the other constructs, *pPLT2-Δbox1;2* did not complement the double mutant whatsoever (Figure 5K), suggesting that BOX1 and BOX2 also function synergistically during early embryogenesis and can to a degree compensate for each other’s absence. It thus becomes apparent that both CNSs are required for early embryogenesis.

### *pPLT1/2* BOX regions exhibit enhanced chromatin accessibility in meristematic tissues

**Figure 6:**
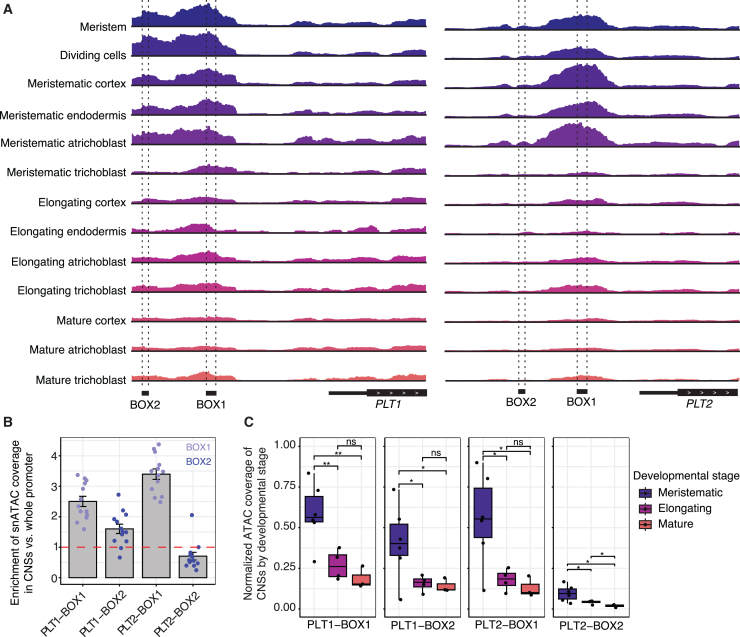
BOX1 and BOX2 reside within accessible chromatin domains. **(A)** snATAC-seq coverage of 13 cell types from an *Arabidopsis* seedling atlas (Baumgart et al., 2025) across the promoter regions of *PLT1* (left) and *PLT2* (right). Coverage tracks are normalized to the maximum value within each promoter (−2 kb to 500 bp of the start codon). **(B)** Fold change in open chromatin coverage for each conserved BOX across all cell types in relation to the average coverage across the whole promoter. Each dot represents a cell type shown in **(A)**. All BOXes had distributions significantly different from the average value (fold change of 1) according to one-tailed *t*-tests (left to right: *p* = 1.11e−6, *p* = 0.002, *p* = 9.12e−9, and *p* = 0.038). **(C)** Distributions of normalized snATAC-seq coverage within conserved BOXes by the developmental stage of the cell types. Coverage was normalized as described in **(A)**. Corresponding Bonferroni-corrected *p* values from *t*-tests are ∗∗*p* < 0.01, ∗*p* < 0.05, and *p <* 1.0 (ns).

Finally, we set out to determine the mechanism through which these CNSs act. We first considered whether the *pPLT2* BOX regions were transcribed, thereby potentially acting as *cis*-regulatory long non-coding RNAs (lncRNAs) involved in, for instance, chromatin looping or recruitment of chromatin modifiers (Yang et al., 2023). In *Arabidopsis*, we did not detect conclusive transcription within the *pPLT2* BOX regions (Supplemental Figure 10), suggesting that these CNSs are not part of a lncRNA. We next examined whether BOX regions could form DNA G-quadruplex structures, which could potentially regulate *PLT2* transcription through binding of specific proteins (Griffin and Bass, 2018). Using QGRS Mapper (Kikin et al., 2006), we found no canonical G_3_L_1-7_ or G_2_L_1-4_ motifs, previously identified in *Arabidopsis,* in either CNS (Mullen et al., 2010). We thus concluded that they are unlikely to form G-quadruplex structures. To further investigate the regulatory landscape of BOX1 and BOX2, we analyzed chromatin accessibility across these regions using a previously published single-nucleus Assay for Transposase-Accessible Chromatin sequencing (snATAC-seq) seedling atlas of *Arabidopsis thaliana* (Baumgart et al., 2025). Visualization of accessible chromatin regions across 13 cell types revealed that both BOX regions in *pPLT1* and *pPLT2* exhibited strong and specific accessibility in meristematic cells compared with elongating and mature developmental stages (Figure 6A). To quantify this enrichment, we compared the snATAC-seq coverage in each BOX region to the average coverage across the entire *PLT* promoter for each cell type (red dashed line; Figure 6B). This analysis showed significantly enriched open chromatin in both BOXes of *pPLT1* and in BOX1 of *pPLT2* (Figure 6B). Although *pPLT2* BOX2 was somewhat accessible, its accessibility was lower than the promoter average (Figure 6A and 6B). We were then interested in measuring the degree of cell-type enrichment, specifically between meristematic, elongating, and mature developmental stages. We normalized the ATAC-seq data across cell types to assess differences in chromatin accessibility across developmental stages. Meristematic cell types consistently exhibited significantly greater accessibility in both BOX regions compared with elongating and mature cell types (Figure 6C). We then asked whether BOX accessibility was also enriched in other species. To this end, we analyzed snATAC data from *Arabidopsis lyrata*, *Capsella rubella*, and *Brassica oleracea* seedlings upstream of two *PLT1/2* paralogs each. However, in these species, the limited number of nuclei per cell type precluded developmental or cell-type-specific comparative analyses. We therefore generated pseudo-bulk profiles by aggregating data from all cells. In all three species, specific accessibility enrichment was consistently observed at BOX1 (Supplemental Figure 11). Accessibility at BOX2 was more variable, reflecting the data from *Arabidopsis* (Figure 6 and Supplemental Figure 11). The preferential openness of BOX1 and BOX2 in *Arabidopsis* and other Brassicaceae species suggests that they might be bound by TFs.

### BOX1 and BOX2 act as PLT-directed (auto)regulation hubs

**Figure 7:**
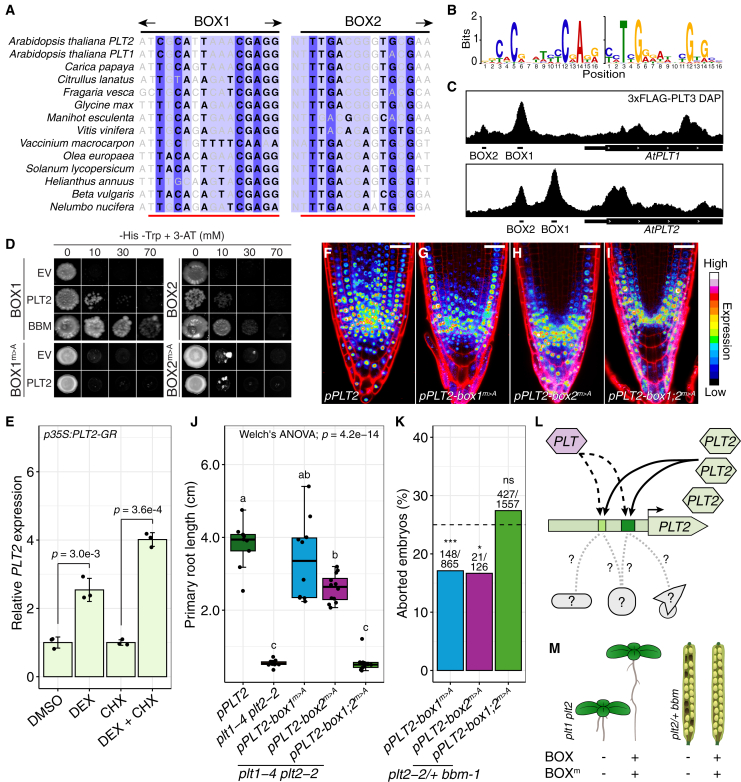
PLT2 activates BOX1 and BOX2 through direct binding to PLT-binding sites. **(A)** Multiple sequence alignments of the BOX1 and BOX2 PLT-binding sites in eudicot species. The blue shading scales with bit score, i.e., the importance of a position in the PLT-binding motif. Bases are grayed out if they do not match the motif at this position. “N” indicates a base not included in our definition of BOX2. Red lines indicate A-substitution sites. **(B)** The canonical PLT-binding motif (right) and reverse complement (left) as described previously, showing bit score per position (Kerstens et al., 2024). **(C)** PLT3 DAP-seq coverage upstream of *AtPLT1* and *AtPLT2*. The *y* axes are equal between the two tracks. **(D)** Yeast one-hybrid assay of pPLT2 BOX1, BOX2, and A-substituted versions over a 3-AT concentration range. EV, empty vector. **(E)** Relative expression (±SD) of endogenous *PLT2* in the *p35S::PLT2-GR* background after 4 h of DMSO, DEX, CHX, or DEX + CHX treatment (*n* = 3). The *p* values are from one-tailed *t*-tests. **(F–I)** Confocal images of 4-dpg PI-stained root tips of *pPLT2::PLT2-YFP* and motif-A-substituted variants. *pPLT2-box1;2*^*m>A*^ is in the *p35S::PLT2-GR* background (uninduced); the others are in Col-0. The YFP signal is shown in the “16 color LUT”. Scale bars, 30 μm. **(J)** Primary root length at 11 dpg in complemented *plt1-4 plt2-2* lines with Games–Howell *post hoc* tests. **(K)** Embryo lethality in offspring of selfed *plt2-2+ bbm-1* mutants complemented with A-substitution constructs. The dashed line at 25% shows the expected embryo lethality level according to Mendelian segregation of the *plt2-2 bbm-1* genotype. Numbers indicate aborted and total embryos. Statistical groups were derived from Bonferroni-corrected one-tailed *z*-tests (abortion <25%). Corresponding *p* values are ∗∗∗*p =* 1.25e−7, ∗*p =* 0.046, and *p <* 1.0 (ns). **(L)** Proposed model describing autoregulation of *pPLT2* through BOX1 and BOX2, in which *pPLT2* is activated by binding of PLT2 to both CNSs, likely redundantly with other PLTs (dashed black arrows). Other TFs are expected to co-regulate *pPLT2* (dotted gray lines). **(M)** Absence of both BOX regions or PLT-binding motifs (BOX^m^) from *pPLT2* compromises *PLT2* expression and its ability to rescue the developmental defects of *plt1 plt2* and *plt2/+ bbm*.

Embedded within both BOX1 and BOX2 was a sequence pattern that resembled an ANT-like motif (Figure 7A and 7B), which has been shown to be bound by all PLTs (Santuari et al., 2016; Kerstens et al., 2024). We therefore hypothesized that the BOXes are subject to PLT-directed (auto)regulation. Using our previously generated PLT3 DNA Affinity Purification sequencing (DAP-seq) data from root DNA (Kerstens et al., 2024), we observed peaks coinciding with the CNSs in both *pPLT1* and *pPLT2* (Figure 7C). We noticed that, in accordance with the fact that the ANT-like motif of *pPLT1* BOX2 contained an adenine at a conserved guanine site, PLT3-bound DNA at this site was only weakly enriched (Figure 7C). We also performed a PLT2 DAP–qPCR on plasmid DNA (pDAP–qPCR) containing wild-type *pPLT2* and again observed binding of PLT2 to both *pPLT2* BOXes (Supplemental Figure 12A and 12B), which was reduced when we used a plasmid template containing A substitutions of the putative PLT-binding motifs (*pPLT2-box1;2*^*m>A*^; Supplemental Figure 12C and 12D). The physical interaction was further corroborated by the fact that PLT2 and BBM bound BOX1 and BOX2 in a yeast one-hybrid assay, but PLT2 could not bind the A-substituted variants (Figure 7D). Combined, these data demonstrate that PLTs, including PLT2 itself, bind *pPLT2* through ANT-like motifs in BOX1 and BOX2.

We then proceeded to ask whether PLT2 could regulate its own expression, thus constituting an autoregulatory feedback loop. First, we quantified *PLT2* transcript abundance in a dexamethasone (DEX)-inducible *p35S::PLT2-GR* line. Four hours after DEX induction, endogenous *PLT2* expression increased, and this also occurred in the presence of the protein synthesis blocker cycloheximide (CHX), confirming that the enhancement effect was direct (Figure 7E). In addition, we observed enhanced expression of transgenic *PLT2-YFP* after induction when we introduced a translational reporter fusion with full-length *pPLT2* in the same background to quantify expression of the transgene (Supplemental Figure 13A). A dual luciferase transactivation assay in leaves of tobacco (*Nicotiana benthamiana*) further confirmed that PLT2 can activate its own promoter (Supplemental Figure 13B and 13C). Notably, whereas loss of BOX2 did not affect transactivation capacity, loss of BOX1 or both CNSs repressed expression of *PLT2* (Supplemental Figure 13C). It remains unclear whether this is caused directly by PLT2-dependent repression, indirectly by intermediary TFs, or by loss of PLT2-dependent activation of *pPLT2*.

If PLT2 binds to PLT-binding motifs inside BOX1 and BOX2 and activates expression of its promoter, then disruption of these motifs should affect *PLT2* upregulation and expression patterns. We therefore introduced A-substitution promoter variants (*pPLT2-box1*^*m>A*^, *pPLT2-box2*^*m>A*^, and *pPLT2-box1;2*^*m>A*^) driving PLT2-YFP into our inducible *p35S::PLT2-GR* line. Whereas the PLT2-YFP signal from *pPLT2* increased 6 h after DEX induction in the meristematic zone of the RAM, signals from *pPLT2-box1*^*m>A*^ and *pPLT2-box1;2*^*m>A*^ did not increase (Supplemental Figure 13D). Induction of PLT2 was still able to enhance the PLT2-YFP signal when driven from *pPLT2-box2*^*m>A*^, however (Supplemental Figure 13D). Thus, on the whole-tissue level, only the PLT-binding motif in BOX1 is required for PLT-directed (auto)regulation.

We next studied the expression patterns of these lines in more detail. We observed that the basipetal PLT2-YFP gradient of *pPLT2-box1*^*m>A*^ was shorter than that of the wild-type promoter and that the signal was faint or absent in distal columella cells, especially within the two central files (Figure 7F and 7G). In the *pPLT2-box2*^*m>A*^ reporter, expression was intact in the columella but absent from the non-stem cell vasculature (Figure 7H). A substitutions of both motifs generated a pattern that shared the properties of both *pPLT2-box1*^*m>A*^ and *pPLT2-box2*^*m>A*^, i.e., lack of vascular expression and reduced central columella expression (Figure 7I), suggesting a modular effect of the PLT-binding motifs in BOX1 and BOX2 on *pPLT2* activity. Importantly, the PLT2-YFP domains of *pPLT2-box1*^*m>A*^ and *pPLT2-box2*^*m>A*^ resemble those of *pPLT2-Δbox1* and *pPLT2-Δbox2*, respectively, but this parallel does not hold true for *pPLT2-Δbox1;2* and *pPLT2-box1;2*^*m>A*^. It thus appears that the PLT-binding motifs in BOX1 and BOX2 are central determinants of the typical and functionally important expression domains in the vasculature and the central columella, but that other BOX elements redundantly regulate *pPLT2* activity in other root cell types.

We then asked whether the expression patterns of the A-substitution lines also compromised the ability to rescue the *plt1-4 plt2-2* and *plt2-2 bbm-1* mutants. Like *PLT2-YFP* expressed from *pPLT2-Δbox1*, expression from *pPLT2-box1*^*m>A*^ complemented the *plt1-4 plt2-2* mutant, not differing from *pPLT2* (Figure 7J). *pPLT2-box2*^*m>A*^*::PLT2-YFP* partially rescued the short root phenotype, mimicking *pPLT2-Δbox2* (Figure 7J). The promoter with A-substituted PLT-binding motifs in both BOX regions was not able to rescue *plt1-4 plt2-2* (Figure 7J). In the *plt2-2/+ bbm-1* mutant background, *PLT2-YFP* driven from *pPLT2-box1*^*m>A*^ and *pPLT2-box2*^*m>A*^ slightly rescued the embryo-lethality phenotype, but to a far lesser extent than the CNS deletion lines (Figure 7K). Again, *pPLT2-box1;2*^*m>A*^ failed to complement the double mutant (Figure 7K). Our data indicate that the autoregulatory PLT-binding motifs in BOX1 and BOX2 are functionally required to regulate *PLT2* expression in roots and/or embryos (Figure 7L and 7M).

## Discussion

Here, we revealed the presence of CNSs upstream of angiosperm *PLT* genes (Figure 1) and uncovered the existence of two deeply conserved eudicot-specific non-coding elements in *PLT1/2* promoters (Figure 2). We demonstrated that these two “BOX” regions are required for wild-type *PLT1/2* expression levels in *Arabidopsis* root tips (Figure 3) and that loss of one or both BOXes alters the *PLT2* expression pattern, which is established during embryogenesis (Figure 4). Furthermore, the altered *PLT2* expression patterns can rescue *plt1 plt2* and *plt2 bbm* double mutants to various degrees, but not if both CNSs are absent (Figure 5). Finally, we show that each BOX resides in accessible chromatin and participates in an autoregulatory feedback loop that shapes *PLT2* expression (Figures 6 and 7).

Although we performed functional studies on only the two most conserved CNSs identified in our analysis, the existence of multiple upstream motifs in each PLT clade strongly points toward extensive CNS-mediated regulatory control. In addition to upstream sequences, this principle likely extends to other genomic regions. For instance, the third intron of *AtPLT3* was shown to contain an enhancer element required for expression in young flowers that is bound by MONOPTEROS, APETALA1, and LEAFY (Kaufmann et al., 2010; Winter et al., 2011; Yamaguchi et al., 2013; Krizek, 2015). Importantly, the specificity of motifs, such as the monocot-specific presence of PLT5 #1–#3 and the Brassicales-specific absence of BBM #1–#3 (Supplemental Figure 2), could point to plant-lineage-specific expression behavior across cell and tissue types. We want to emphasize, however, that sharing a conserved CNS does not necessarily imply that homologs are expressed identically. Even in *Arabidopsis*, *pPLT1* and *pPLT2* have different expression patterns, with only *PLT2* being expressed in the zygote and also much more strongly expressed in the root tip columella than *PLT1* (Galinha et al., 2007; Kerstens et al., 2024).

Because multiple lines of evidence suggest that BOX1 and BOX2 modulate *PLT2* expression through direct binding of PLTs, we find it reasonable to assume that they serve as TF-binding hubs and act as enhancers. However, important questions about the mechanistic action of these CNSs on expression remain unanswered. First, why are they so long? TF-binding motifs are short (∼5–20 nt) and typically degenerate, suggesting that the interplay of dozens of competing factors could potentially converge on these CNSs to conditionally tune development. For instance, enhancer region activity in tobacco leaves varied greatly across different light conditions, acting either cooperatively or independently (Jores et al., 2024). Our reporter lines showed that BOX deletion conditionally (i.e., cell-type specifically) disrupted expression and did not simply reduce overall promoter activity, suggesting that cell-type-specific factors bind to these CNSs. Given that substitution of the PLT-binding motif largely mimics complete BOX loss, it is unclear how the rest of the CNSs contribute to promoter activity. Perhaps binding of PLT is a prerequisite for recruitment of cofactors, cooperatively tuning *pPLT1/2* activity. In agreement with this notion, WOX5 was shown to bind PLT1-3 and BBM *in planta* through intrinsically disordered prion-like domains at their C termini, and this binding was required for transgenic PLT3-mVENUS to alleviate the stem cell niche defects in *plt2 plt3* double mutants (Burkart et al., 2022). In addition, the physical interaction between PLTs and TEOSINTE-BRANCHED CYCLOIDEA PCNA (TCP) TFs is required for their interaction with SCARECROW (SCR), and combined loss of *PLT1*, *PLT3*, *SCR*, and *TCP20* caused RAM arrest (Shimotohno et al., 2018). These findings imply that PLT-mediated TF convergence on the ANT-like motif, perhaps facilitated by proximal sequences in the CNSs, could explain the expression patterns resulting from the A substitutions. Alternatively, the A substitutions might disrupt accessibility of the BOX in general, preventing not only PLT docking but also blocking overlapping or adjacent binding sites. We note that simple expression (pattern) analyses on a single agar medium may be too crude to detect more subtle BOX functions, as multiple internal and external factors may converge on this single phenotypic readout.

Second, why is the distance between the BOX regions and their order conserved in eudicots? The order and spacing of enhancer fragments have recently been shown to conditionally affect enhancer activity, suggesting that relative BOX position is evolutionarily constrained (Jores et al., 2024). However, our *pPLT2-swap::gPLT2-YFP* reporter showed only a minor effect on the *PLT2* expression domain and could complement both *plt1-4 plt2-2* and *plt2-2 bbm-1*, suggesting that their relative position is not as vital as their conservation would suggest. Moreover, the truncated *pPLT2-1.3kb* promoter that lacked BOX2 did not change the expression pattern in comparison to *pPLT2-Δbox2*, suggesting that the enhancing properties of BOX1 remain intact in this context. Our data thus indicate that, under our experimental conditions, the CNSs act as autonomous enhancing units that can function independently of each other. Taking these results together, we identified a series of deeply conserved angiosperm CNSs near master regulators of pluripotency and functionally dissected the role of two of these elements in *Arabidopsis*. Future detailed studies will be required to determine whether BOX1 and BOX2 function similarly in other species, but given that within-TF-family motifs are strongly conserved between even distantly related species (Baumgart et al., 2025), we predict that they constitute a universal mechanism for regulation of eudicot root and embryo development.

## Methods

### Extraction of promoter sequences

Protein sequences of PLT homologs (HOM05D000138 and HOM05M000121) were downloaded from PLAZA5.0 Dicots and Monocots, respectively (Van Bel et al., 2022). For rice and maize, only *O. sativa* ssp. *japonica* and *Zea mays* B73 were included. *Phaseolus vulgaris* and *Theobroma cacao* were not included owing to unavailability of the data. PLT protein sequences between 200 and 800 amino acids were submitted to MAFFT v.7 (Katoh et al., 2017) for multiple sequence alignment in FFT-NS-2 mode with a gap-opening penalty of 1.0. The multiple sequence alignment was trimmed using trimAl v.1.4.rev.15 (Capella-Gutiérrez et al., 2009) with the flags -gt 0.5 and -cons 0.7. A phylogenetic tree was generated with IQ-TREE v.2.2.6 (Minh et al., 2020) in -m test mode with 1000 ultrafast bootstraps (-bb 1000). Branches with <70 bootstrap support were deleted using iTOL v.6.8.2 (Letunic and Bork, 2024). From the resulting trees, clades were defined manually according to *Arabidopsis* homologs. For each PLT clade, truncated and complete promoters of all genes were extracted using bedtools v.2.31.1 (Quinlan and Hall, 2010). Truncated promoters were defined as the shortest of either 20 kb upstream of the annotated PLT CDS oruntil the CDS of the first upstream gene. Full promoters were defined as 20 kb upstream of the PLT CDS or until the CDS of another PLT homolog, to exclude overlapping tandem repeats. All promoters were required to be >50 bp in length.

### Detection and selection of conserved elements

The truncated promoter sequences of each PLT clade were submitted to MEME v.5.5.4 (Bailey et al., 2015) to detect conserved motifs, and this analysis was performed separately for the PLAZA monocot and eudicot datasets. MEME was run in “zoops” mode, detecting motifs of 15–100 bp present in at least 15 promoters. Significant motifs (E < 0.05) were manually curated to be highly conserved sequence-wise (long stretches of bit scores >1), to not have non-specific hits (e.g., matching in repetitive sequences or matching many more locations in the promoters than the position-specific scoring matrix was originally based on), and to not represent repetitive regions (motifs consisting of 1 or 2 nt repeated throughout the whole motif). Using FIMO (Grant et al., 2011), the full promoters of both monocots and eudicots were re-scanned for the presence of selected monocot and eudicot motifs in each PLT clade. Only hits (motif × promoter combinations) with a *q* < 0.01 and in the same orientation as the gene were selected. In addition, to remove false positive hits, a motif was required to be present in at least 10% of the respective clade (phylogenetic clade: monocots, asterids, or rosids; in combination with the four PLT clades), removing 32 of 1802 hits. This threshold was not applied to early diverged eudicots and early diverged angiosperms because of the low sampling depth in these clades. The phylogenetic species tree was adapted from PLAZA5.0 (Van Bel et al., 2022) and annotated with iTOL v.6.8.2 (Letunic and Bork, 2024). Downstream analysis and visualization were performed in R (see Supplemental Table 6 for packages).

### Characterization of BOX1 and BOX2

Sequences constituting the upstream *PLT1/2* motifs PLT1/2 #1 (BOX1) and PLT1/2 #3 (BOX2), in addition to the 100-bp flanking sequence on both sides, were extracted from all analyzed PLAZA5.0 eudicot genomes using bedtools v.2.31.1 (Quinlan and Hall, 2010). The BOX sequences and CDSs were aligned with MAFFT v.7.419 (Katoh and Standley, 2013) using --auto settings. BOX regions were defined manually within the multiple sequence alignment using Jalview v.2.11.4.1 (Waterhouse et al., 2009). Pairwise percentage identity calculations between BOX sequences were performed with Clustal Omega v.1.2.4 (Sievers et al., 2011), specifying --distmat and --percent-id.

### Plant materials

*Arabidopsis* ecotype Col-0 was used as the wild type. *plt1-4 plt2-2* and *plt2-2 bbm-1* have been described previously (Aida et al., 2004; Kerstens et al., 2024). *plt1 plt2-cr* and the four *ΔpPLT1*/*ΔpPLT2* alleles were generated in the Col-0 background (see “cloning”). *pplt2-cr* alleles in the *plt1-cr* background were generated by crossing homozygous *ΔpPLT2* lines to *plt1 plt2-cr*, and *ΔpPLT1* alleles in the *plt2-cr* background were obtained in the same way. *Arabidopsis* plants were grown under long-day conditions (16 h light, 8 h dark) at 22°C under white fluorescent tube lights (plates) or white LED lights (pots). Tobacco plants were grown under the latter conditions.

### Cloning

The transcriptional and translational reporter constructs for full-length *pPLT2*, i.e., *pPLT2::erCFP* and *pPLT2::gPLT2-YFP*, as well as *p35S::cPLT2-GR*, were generated previously in pGreenII0227 backbones that conferred hygromycin resistance (Hellens et al., 2000; Galinha et al., 2007). BOX deletions were generated by first subcloning a PmeI/AvrII-digested 824-bp fragment from *pPLT2::gPLT2-YFP* into pGEM-T Easy 221 containing both elements and subsequent BOX deletion through blunt ligation of Δbox oligos. The native *pPLT2* sequence was replaced by the modified subclone fragments using PmeI and AvrII. The *pPLT2* truncations were made by Gateway cloning. Adenine substitutions were made using site-directed mutagenesis PCR. The translational reporters in the *p35S::cPLT2-GR* background were made in the pGreenII0124 (methotrexate resistant) backbone. *pPLT2-swap::gPLT2-YFP* was generated by performing PCR of five BsaI-flanked amplicons from the original full-length translational reporter construct in pGreenII0124 and inserting annealed BOX1 and BOX2 oligos with fitting overhangs to seamlessly reconstitute the rest of the vector through BsaI digestion and ligation. CRISPR-Cas9 mutagenesis was performed as described in Kerstens et al. (2024) using one single-guide RNA (sgRNA) targeting *PLT1* and *PLT2* and nine sgRNAs targeting the non-coding regions surrounding *pPLT1/2* BOX1 and BOX2. All *Arabidopsis* constructs were transformed into *Agrobacterium tumefaciens* (C58C1.pMP90) and transferred to *Arabidopsis* by the floral dip method (Clough and Bent, 1998). For the yeast one-hybrid assay, annealed BOX1 and BOX2 oligos with or without A-substituted PLT binding motifs were cloned into the entry vector pDONR221 (Invitrogen) and then into the bait destination vector pMW#2 (Addgene #13349) or pINT1-HIS3NB (NovoPro #V005419), respectively. *cPLT2* was cloned into pDONR221 and then into the prey destination vector pDEST22. For the dual luciferase assay, *pPLT2*, *pPLT2-Δbox1*, *pPLT2-Δbox2*, and *pPLT2-Δbox1;2* were cloned into pGEM-T Easy 221 (Invitrogen) and subsequently into pGreen-LUC68 (Adrian et al., 2010). The *PLT2* CDS was cloned into pGWB41 (Nakagawa et al., 2007) from pGEM-T Easy 221. The DAP vector pSPUTK-GG 3×FLAG-cPLT2 was made by amplifying *cPLT2* from Col-0 root cDNA and combining the amplicon with pICSL30005 (Addgene #50299; 3×FLAG) and pSPUTK-GG (Kerstens et al., 2024) through Golden Gate cloning (Engler et al., 2008). Oligonucleotides used for cloning and genotyping are listed in Supplemental Table 7.

### Confocal microscopy

Confocal microscopy was performed with a ZEISS LSM 710. erCFP, YFP, and PI were excited with 458-, 488-, and 543-nm lasers, respectively, and emission was detected in the 460–530, 500–530, and 600–660 nm range, respectively. RAM expression was studied in 4-dpg seedlings grown on ½ germination medium (½ GM: 1% sucrose, 0.8% plant agar, 2.2 g/l Murashige and Skoog medium + vitamins, and 0.5 g/l MES [pH 5.8]; Duchefa) with 50 mg/l ampicillin (Duchefa) using variable settings. Signal intensity was visualized with the “16 color LUT” in Fiji (Schindelin et al., 2012).

### Mutant and complementation assays

Primary root growth of *ΔpPLT1* and *ΔpPLT2* alleles and *plt1-4 plt2-2* complementation lines was tracked in seedlings grown on plates with ½ GM + 50 mg/l ampicillin by marking the position of the tip over time. The roots were then scanned and traced in Fiji (Schindelin et al., 2012). RAM length was determined in 7-dpg seedlings by measuring the average distance between the QC and the first elongating cortex cell on both sides of the root. mPS-PI staining was performed on root tips of 5-dpg seedlings as described previously (Truernit et al., 2008). Seed abortion in *plt2-2/+ bbm-1* was counted in maturing siliques of 5- to 7-week-old plants as described in Kerstens et al. (2024). All complementation lines were homozygous for a single transfer DNA locus (as inferred from 3:1 segregation of the T_2_ progeny) and analyzed in T_3_ or later generations.

### G-quadruplex analysis

*pPLT2* BOX1 and BOX2 sequences were scanned for the G-quadruplex motifs G_3_L_1-7_ and G_2_L_1-4_ using QGRS Mapper (Kikin et al., 2006), specifying “Min G group” as 3 or 2, respectively, and “Loop size” as 1 to 7 or 1 to 4, respectively.

### Yeast one-hybrid assay

Competent PJ69-4α yeast was transformed with the linearized pMW#2 (XhoI) or pINT1-HIS3NB vector (NsbI) containing bait DNA and selected on −His dropout medium (Sigma-Aldrich). Transformed yeast was mated overnight on YPAD medium with the PJ69-4A strain containing *pDEST22 cPLT2* and selected on −His −Trp dropout medium or, alternatively, directly transformed with this construct and selected in the same way. Interactions were scored after 3 days of incubation at 28°C at a range of 3-AT (Sigma-Aldrich) concentrations.

### RNA extraction and RT–qPCR

RNA was extracted from 3-dpg (*p35S::PLT2-GR*), 5-dpg (Col-0, *plt2-2*, *ΔpPLT1*, and *ΔpPLT2*), or 4-dpg (*pPLT2::gPLT2-YFP* in *p35S::PLT2-GR*) root tips grown in ½ MS (½ GM without sucrose) plates on top of a nylon mesh. Inductions were performed by transferring the mesh to ½ MS supplemented with 0.1% DMSO (Sigma-Aldrich), 10 μM DEX (Sigma-Aldrich), 10 μM CHX (Sigma-Aldrich), or 10 μM DEX + CHX. Total RNA was extracted from the samples with the Spectrum Plant Total RNA Kit (Sigma-Aldrich) according to the manufacturer’s instructions. cDNA was synthesized with the RevertAid reverse transcriptase system (Fermentas) using oligo(dT)18 primers, and expression was quantified using SYBR Green in the Bio-Rad CFX Connect Real-Time PCR detection system using two or three technical replicates per biological replicate. Endogenous *PLT1* expression was amplified with PLT1-qPCR primers, *PLT2* with PLT2-qPCR primers, and *PLT2-YFP* with PLT2-YFP primers, and expression within the promoter was assayed with lncRNA R1–R8 (Supplemental Table 7). Expression was normalized against that of the housekeeping gene *UBC21* (AT5G25760).

### PLT2-YFP signal induction assay

Quantitative PLT2-YFP measurements were performed with 4-dpg seedlings grown on ½ GM with ampicillin on top of a nylon mesh as described above. Imaging was performed at 100× magnification using identical confocal settings for all lines and experiments. Confocal images were analyzed in Fiji. Signal intensity was quantified by rotating the roots vertically (tip down) and then determining the integrated density of the YFP channel in a 208 by 554 μm rectangular selection from the root tip after subtraction of the background signal.

### Dual luciferase assay

The dual luciferase assay was performed as described previously (Díaz-Triviño et al., 2017). In brief, the second youngest leaves of 2-week-old tobacco plants were infiltrated with an *A. tumefaciens* (C58C1.pMP90) suspension containing *p35S*::*RENILLA*, p19, *pGreen-LUC68 pPLT2*, and *p35S::YFP* (negative control) or *p35S::cPLT2-YFP* in a 0.4:1:1:1 ratio. Four days after infiltration, luminescence was quantified in cell extracts of three infiltrated leaf disks per leaf (technical replicates) with the GloMax96 microplate luminometer (Promega).

### Plasmid DAP–qPCR

The pDAP–qPCR was performed as described for the DAP-seq in Kerstens et al. (2024) with several modifications. Instead of *Arabidopsis* gDNA, *pGII0124 pPLT2::gPLT2-YFP* and *pGII0124 pPLT2-box1;2*^*m>A*^*::gPLT2-YFP* pDNAs were sonicated to a fragment size of 100–400 bp. Approximately 100 pg of sonicated pDNA was directly used in a 3×FLAG-PLT2 and 3×FLAG-GFP DAP. 3×FLAG-protein-pDNA complexes were eluted from anti-FLAG M2 magnetic beads (Sigma-Aldrich) by incubation at 98°C in 100 μl TE buffer. The eluates were then analyzed by qPCR as described above using pDAP–qPCR primers R1–R6 (Supplemental Table 7). Fold enrichment was calculated by normalizing to R6 (*NPTI*) in the backbone of the plasmid.

### Open chromatin around PLT boxes with snATAC-seq

We extracted ATAC reads from 2000 bp before the start codon to 500 bp after the start codon (hereafter referred to as the promoter region) of *PLT1* and *PLT2* using the *Arabidopsis* seedling snATAC-seq atlas (Baumgart et al., 2025). In total, we used data from 13 different cell types comprising meristematic, elongating, and mature cell types. We used deepTools v.3.5.4 (Ramírez et al., 2016) to create BigWig files for each cell type with the program bamCoverage, considering genome size, using a bin size of 1, ignoring duplicates, and using “PRGC” as the normalization method. The resulting BigWig files are available at Zenodo (https://doi.org/10.5281/zenodo.15029176). For visualization, we used pyBigWig from deepTools to read the BigWig file and custom scripts to plot the tracks. For each paralog, the snATAC tracks were normalized to the highest value across the 13 cell types in the promoter region. We also calculated the mean value across the whole promoter region and then compared the mean to the value within each BOX for each cell type individually. To evaluate cell-type openness by developmental stage, we separated the data into meristematic, elongating, and mature cell types for each PLT-BOX combination. We explored chromatin accessibility in *A. lyrata*, *C. rubella*, and *B. oleracea* using a pseudo-bulk approach by aggregating data from all cells in their respective snATAC seedling atlases (Baumgart et al., 2025). ATAC coverage was examined in the promoter regions of two *PLT1/2* paralogs in each species, and the openness of each BOX relative to that of the whole promoter was determined as described above.

### Data availability

In-house scripts used for motif identification, as well as the *PLT1/2* BOX and CNS sequences, can be accessed on GitHub (https://github.com/merijnkerstens/plt-cns/). snATAC-seq tracks for 10 kb upstream and downstream of the BOX regions can be accessed through a Zenodo repository (https://doi.org/10.5281/zenodo.15029176).

## Funding

This research was funded by the 10.13039/501100003246Nederlandse Organisatie voor Wetenschappelijk Onderzoek (GSGT.2019.019 to M.K.). The work conducted by the Joint Genome Institute was supported by the Office of Science of the US Department of Energy (Contract No. DE-AC02-05CH11231). Open access funding was provided by Wageningen University & Research.

## Acknowledgments

We thank Max Broers, Hugo Hofhuis, Luca Santuari, Michael Schon, Gertjan Wesselink, and Qian Xun for assisting with experiments and data analysis. We thank Renze Heidstra and Richard Immink for critical reading of the manuscript and helpful suggestions. No conflict of interest is declared.

## Author contributions

Conceptualization, V.W.; methodology, M.K., Y.B., R.O., and V.W.; software, M.K. and Y.B.; formal analysis, M.K., Y.B., A.M.-C., C.R., P.W., L.A.B., G.S.-P., and V.W.; investigation, M.K., Y.B., A.M.-C., P.W., L.A.B., G.S.-P., and V.W.; writing, M.K., Y.B., A.M.-C., R.O., and V.W.; editing, B.S.; visualization, M.K., Y.B., A.M.-C., and V.W.; supervision, V.W.; project administration, V.W.; funding acquisition, M.K.

## Supplemental information

Data S1. Supplemental Tables 1–7

Document S2. Article plus supplemental information
