# Supplementary material for: Two deeply conserved non-coding sequences control PLETHORA1/2 expression and coordinate embryo and root development
Source: Plant Commun. 2025 Jul 29;6(10):101466. doi: 10.1016/j.xplc.2025.101466 (PMC12545816; doi:10.1016/j.xplc.2025.101466)
Supplement: Document S1. Supplemental Figures 1–13 [file mmc1.pdf]

Plant Communications, Volume *6*

Supplemental information

Two deeply conserved non-coding sequences control *PLETHORA1/2* expression and coordinate embryo and root development

Merijn Kerstens, Yvet Boele, Abraham Morales-Cruz, Chris Roelofsen, Peng Wang, Leo A. Baumgart, Ronan O'Malley, Gabino Sanchez-Perez, Ben Scheres, and Viola Willemsen

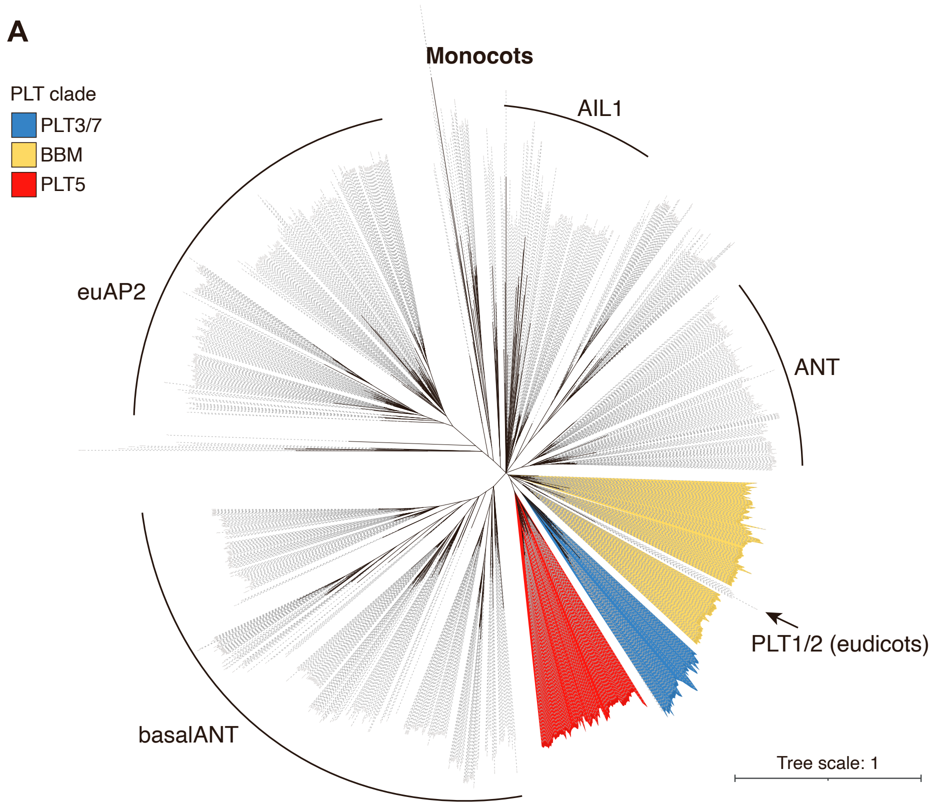

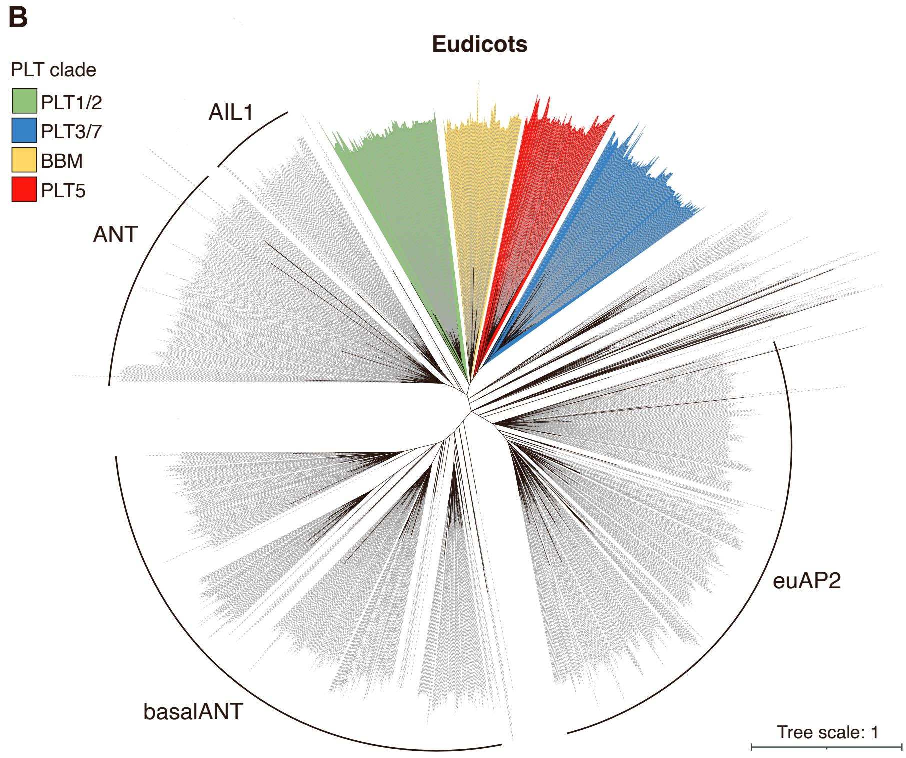

Figure S1. Unrooted phylogenetic trees of the PLAZA5.0 monocots HOM05D000121 (**A**) and eudicots HOM05M000138 (**B**) homology groups. PLT clades are marked in color. Related AP2/EREBP clades, including the euANT-type AIL1 and ANT clades, are indicated. Note that the 8 eudicot PLT1/2 sequences from PLAZA5.0 monocots are nested within the BBM clade in (**A**); PLT1/2 forms a separate clade when more sequences are included in (**B**).

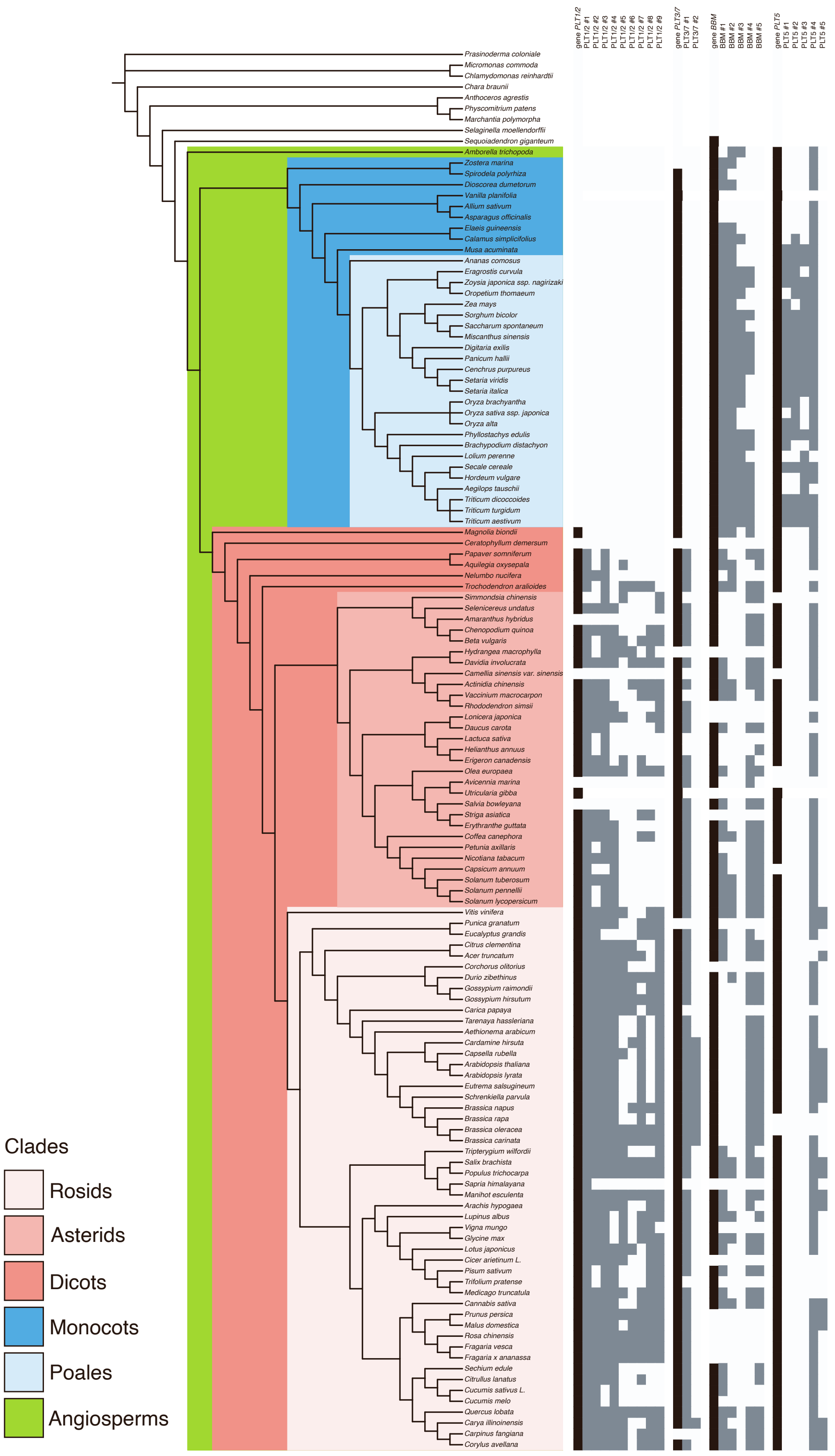

Figure S2. Lineage-specificity of PLT clades and upstream motifs. Ortholog and motif presence is indicated in black and gray, respectively. The species phylogeny is color-coded by angiosperm lineage.

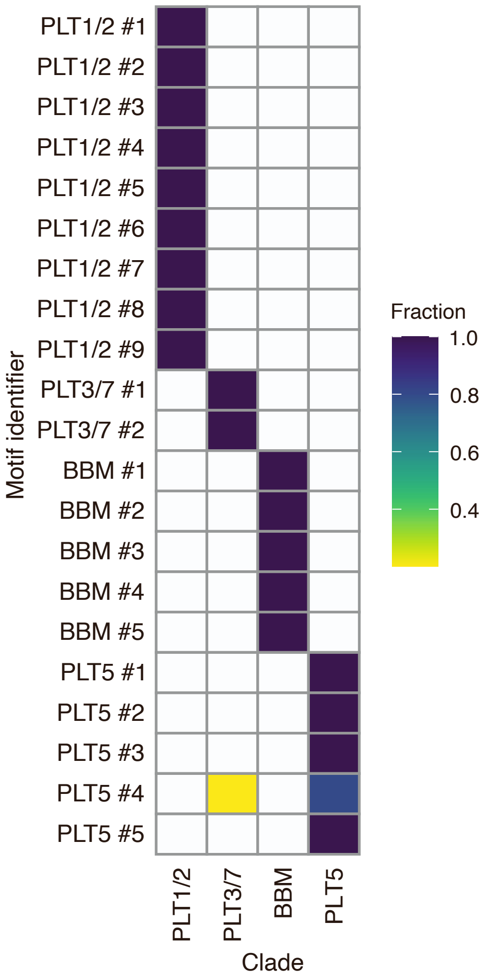

Figure S3. PLT motif clade-specificity. All motifs except PLT5 #4 are specific to a particular PLT clade.

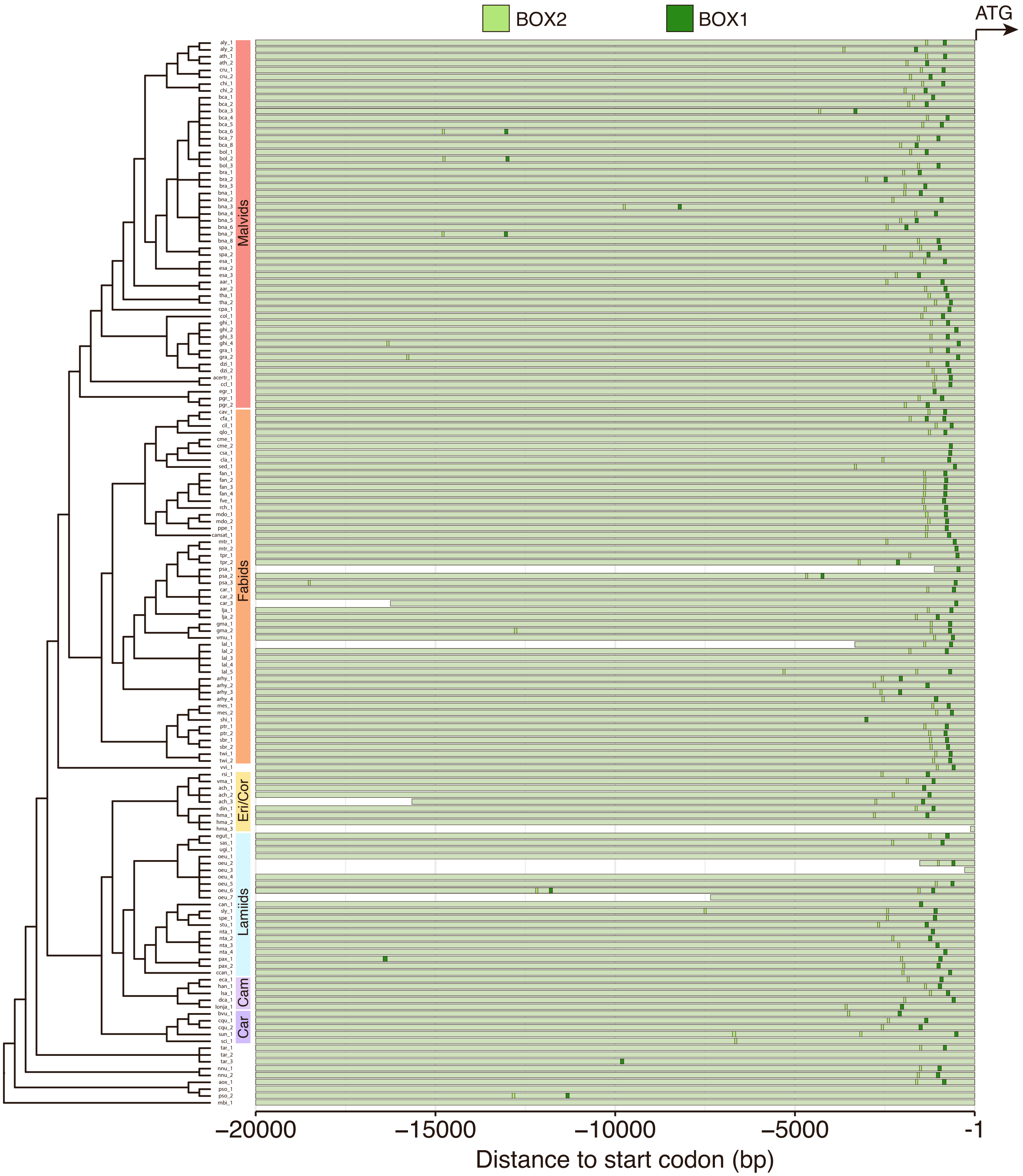

Figure S4. Position of the two *PLT1/2* CNSs relative to the start codon across identified orthologs. BOX1 and BOX2 are portrayed by dark green and light green shading, respectively. Ortholog nomenclature is listed in Table S4. Phylogeny according to species divergence, with major taxa indicated. Lineage abbreviations are: Eri/Cor = Ericales/Cornales, Cam = Campanulids, Car = Caryophyllales.

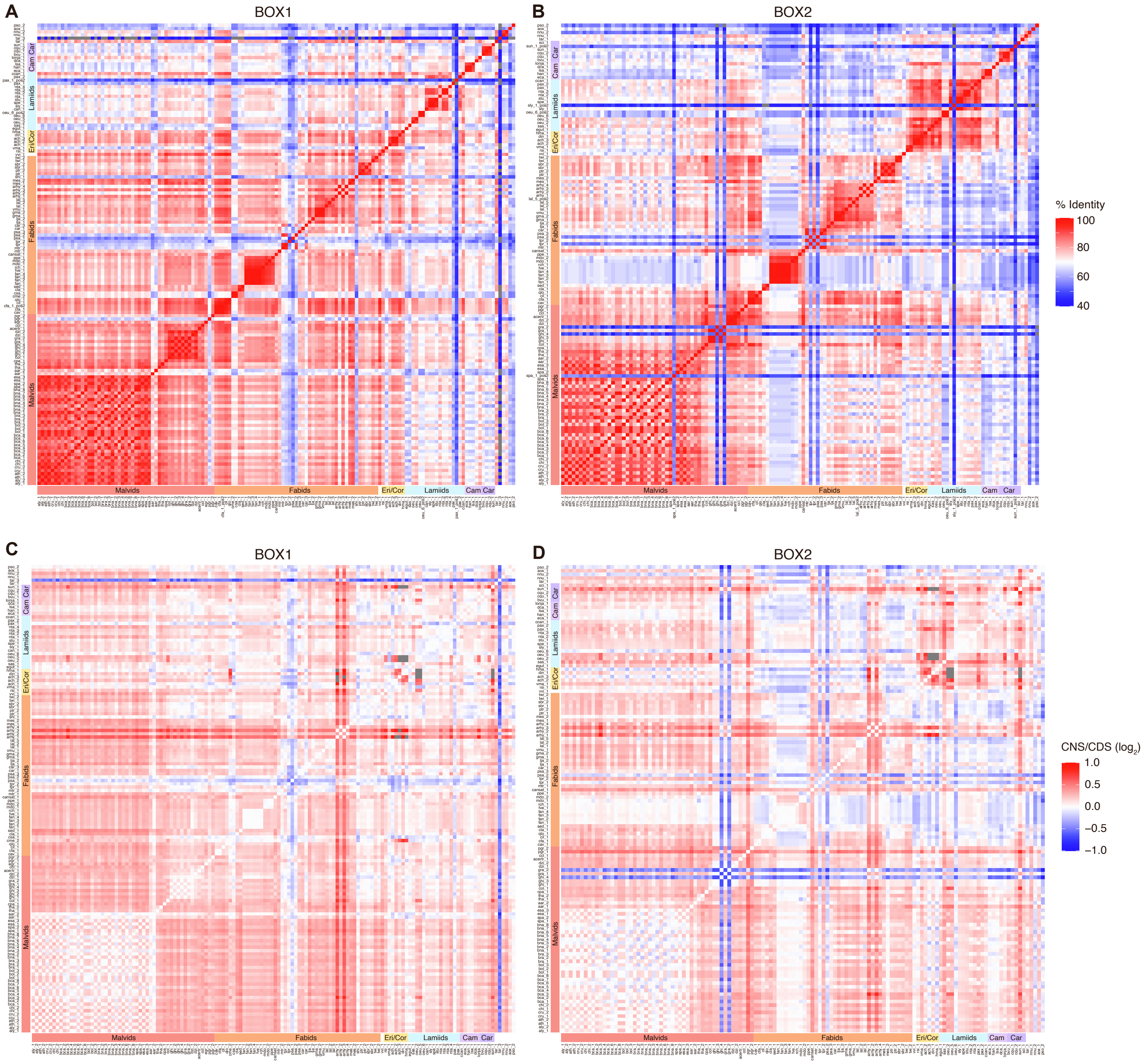

Figure S5. *PLT1/2* CNSs are extremely conserved. (**A**) Reciprocal heatmap of percent identity for BOX1 and BOX2 (**B**) between pairwise comparisons of all identified BOX sequences across all species, with white demarcating the boundary between > 70% (red) or < 70% identity match (blue). If two instances of BOX1 or BOX2 were identified, these sequences are marked with 'pos2'. (**C**) Log_2 enrichment of percent identity between pairwise comparisons of BOX1 or BOX2 (**D**) and the CDS of the respective ortholog. Only the closest instance of BOX1/BOX2 was analyzed. Red and blue indicate higher and lower percent identity of a BOX sequence compared to the CDS, respectively. Sequence/ortholog nomenclature is listed in Table S4. Major eudicot taxa are indicated. Lineage abbreviations are: Eri/Cor = Ericales/Cornales, Cam = Campanulids, Car = Caryophyllales.

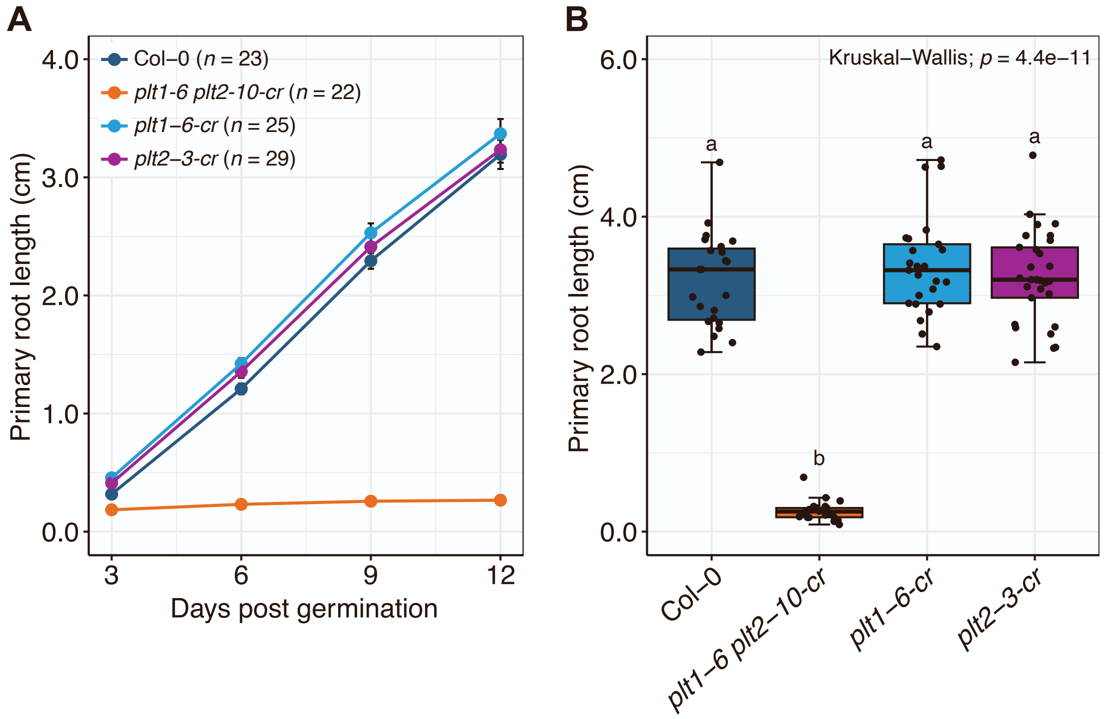

Figure S6. *plt1* and *plt2* single mutant primary root growth is unaffected. (**A**) Time course of primary root growth (± SEM) between wild type, *plt1 plt2-cr*, and *plt-cr* alleles. (**B**) Primary root length at 12 dpg with Dunn's *post hoc* tests. Data are derived from (**A**).

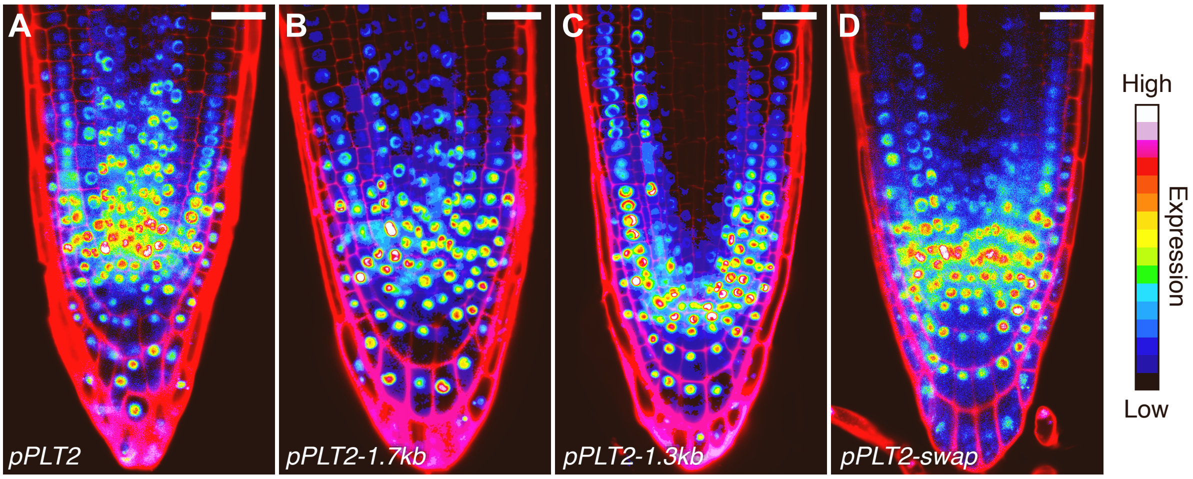

Figure S7. Truncations of *pPLT2* mimic BOX deletion effects. (**A**) PLT2-YFP driven from *pPLT2* and variants (**B-D**) in 4 dpg Col-0 and *plt1-4 plt2-2* (*pPLT2-1.7kb*) root tips stained with PI. PLT2-YFP signal according to the "16 color LUT". Scale bars, 30 µm.

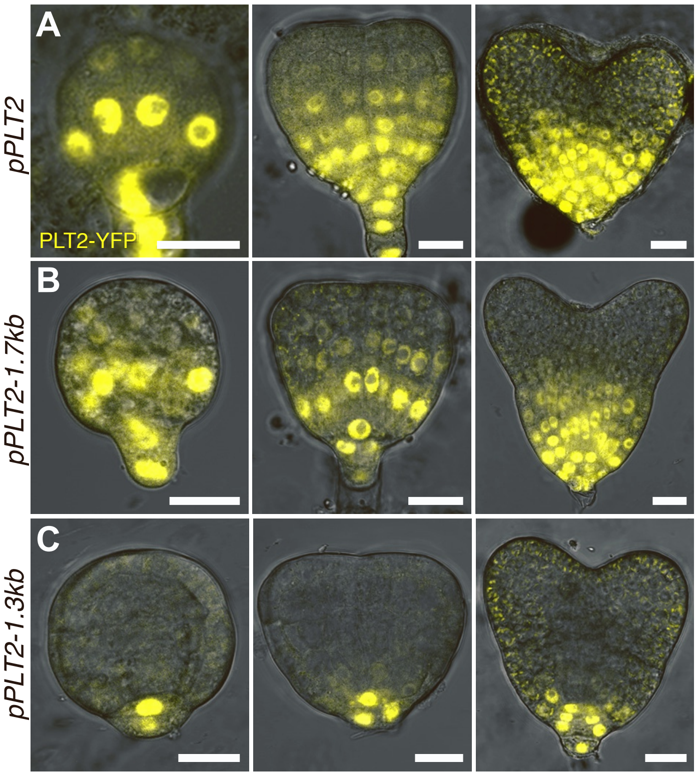

Figure S8. Truncations of *pPLT2* mimic BOX deletion effects. (**A**) PLT2-YFP driven from *pPLT2* and truncation variants (**B-C**) during embryogenesis in Col-0, except for *pPLT2-1.7kb*, which was in the *plt1-4 plt2-2* background. Scale bars, 15 µm.

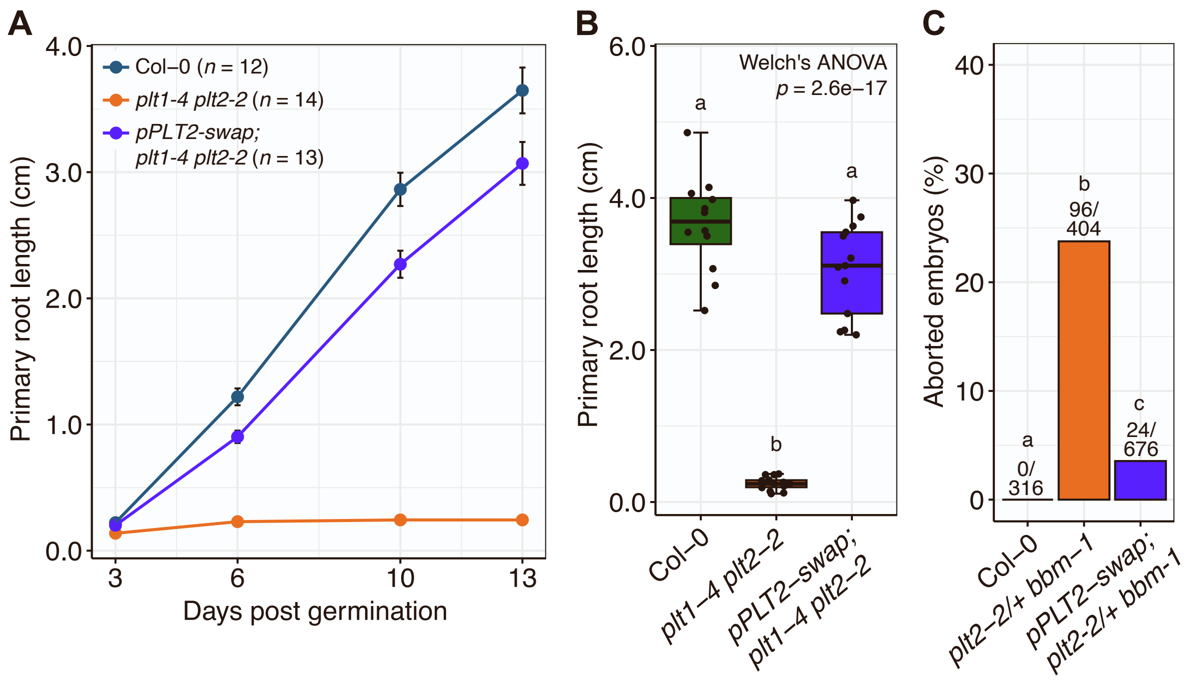

Figure S9. *pPLT2-swap* driving *gPLT2-YFP* rescues *plt1-4 plt2-2* and *plt2-2 bbm-1* double mutants. (**A**) Time course of primary root growth (± SEM) between Col-0 (n = 12), *plt1-4 plt2-2* (n = 14), and *plt1-4 plt2-2* mutants complemented with *pPLT2-swap::PLT2-YFP* (n = 13). (**B**) Primary root length at 13 dpg in *plt1-4 plt2-2* complemented with *pPLT2-swap* with Games-Howell *post hoc* tests. Data are derived from (**A**). (**C**) Embryo lethality in offspring of the indicated genotypes. Numbers are aborted and total embryos, respectively. Statistical groups from pairwise *z*-tests with Yates' continuity and Bonferroni correction.

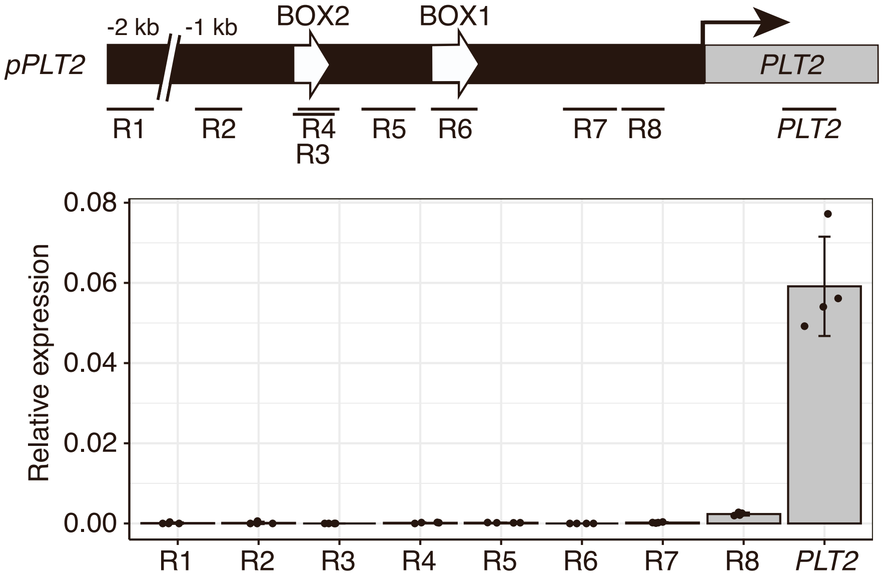

Figure S10. The region containing BOX1 and BOX2 is not expressed in the RAM. Relative expression (± SD) of regions R1-R8 and *PLT2* as determined by qPCR, normalized to the *UBC21* housekeeping gene, in 5 dpg Col-0 root tips. 4 biological replicates were performed.

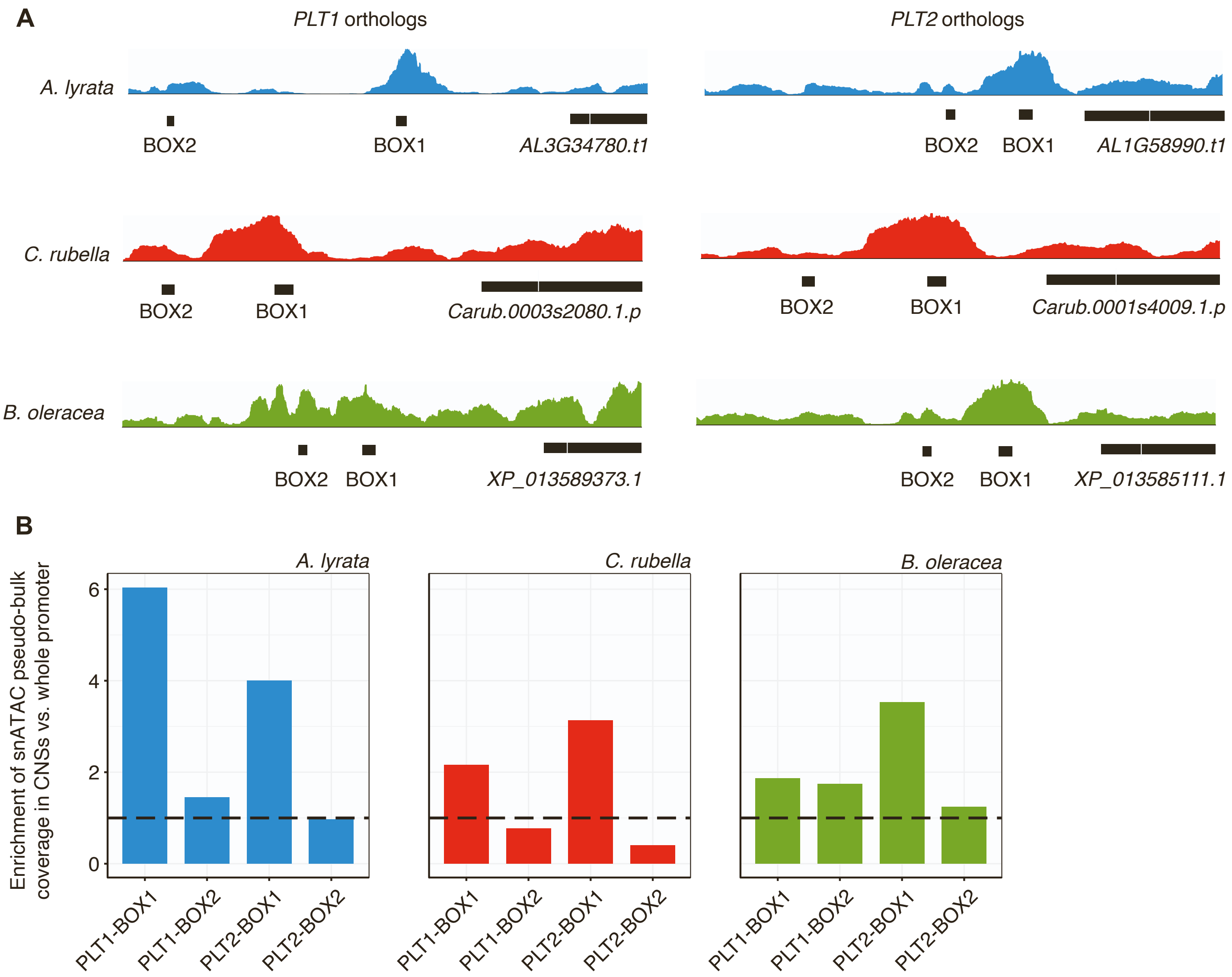

Figure S11. *PLT1/2* BOX regions in *A. lyrata*, *C. rubella* and *B. oleracea* reside in accessible chromatin regions using pseudo-bulk snATAC-seq data. (**A**) Pseudo-bulk snATAC-seq coverage plots of *PLT1* (left) and *PLT2* (right) orthologs. The gene identifiers correspond to *aly_1* (*AL1G58990.t1*), *aly_2* (*AL3G34780.t1*), *cru_1* (*Carub.0001s4009.1.p*), *cru_2* (*Carub.0003s2080.1.p*) in tables S1 and S4. *XP_013589373.1* and *XP_013585111.1* are RefSeq annotation IDs and do not correspond directly to PLAZA5.0 sequences. (**B**) Enrichment compared to whole promoter (dotted line; 1).

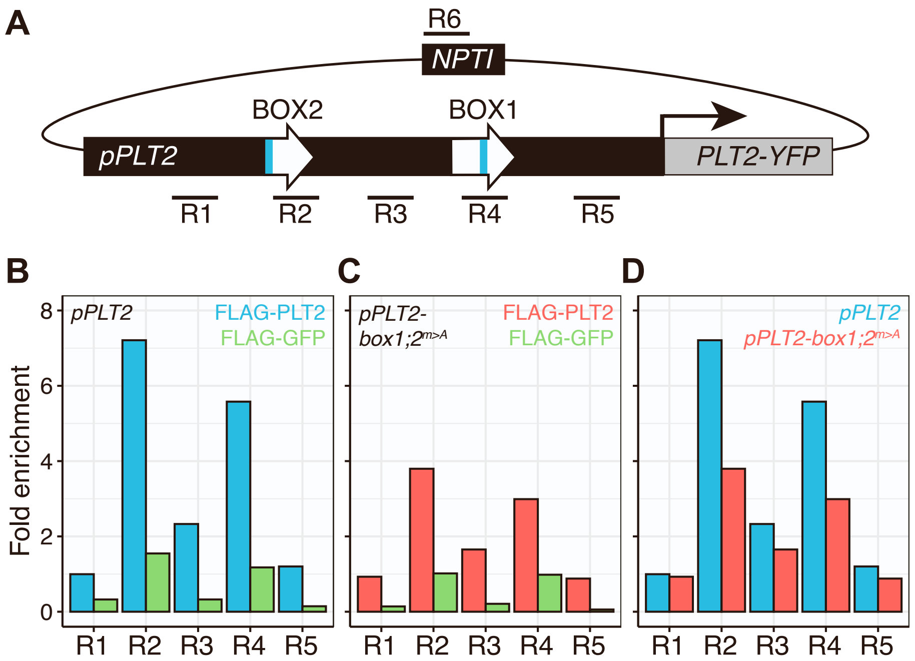

Figure S12. PLT2 binds to the PLT-binding motif in BOX1 and BOX2 in a plasmid DAP. (**A**) Schematic overview of the vector used for pDAP. qPCRs were performed on regions R1-R6, with R2 and R4 corresponding to BOX2 and BOX1, respectively. The PLT-binding motifs inside each BOX are indicated in blue. (**B**) Fold enrichment of regions R1-R5 over R6 in a pDAP-qPCR experiment with 3xFLAG-PLT2 or with 3xFLAG-GFP as negative control, using plasmids containing *pPLT2::PLT2-YFP* or *pPLT2-box1;2*$^{m>A}$*::PLT2-YFP* (**C**). (**D**) Side-by-side comparison of the 3xFLAG-PLT2 pDAP fold enrichment data from panels (**B**) and (**C**).

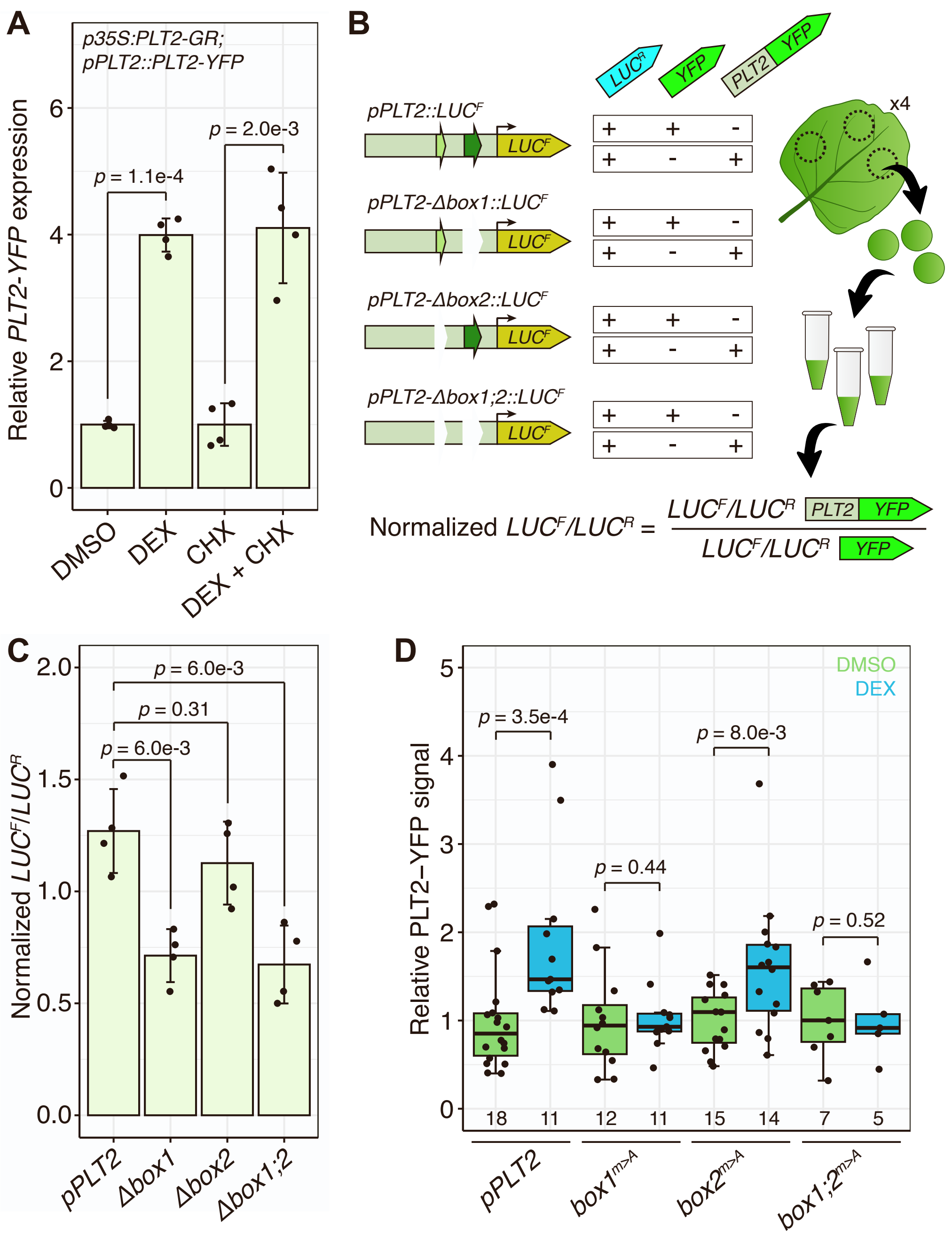

Figure S13. PLT2 activates its own promoter. (**A**) Relative expression (± SD) of *PLT2-YFP* transgene in the *p35S::PLT2-GR* background after 4 hours DMSO, DEX, CHX and DEX + CHX treatment (n = 4). p-values from one-tailed t-tests. (**B**) Experimental setup of the dual luciferase assay. Each promoter variant driving firefly luciferase (LUC^F) was co-infiltrated in tobacco with Renilla luciferase (LUC^R) and either *p35S::YFP* or *p35S:PLT2-YFP*. Extracts from individual leaf disks (3 disks per leaf; technical replicates) of four independent leaves (biological replicates) were used for analysis. (**C**) Ratio of Firefly:Renilla luciferase (± SD) measured in tobacco after infiltration with *pPLT2* promoter variants and *p35S::PLT2-YFP* (n = 4). Values are normalized to the *p35S::YFP* control; values above 1 indicate activation, values below repression. p-values from multiple two-tailed t-tests after Benjamini-Hochberg correction. (**D**) PLT2-YFP signal detected in RAMs of *pPLT2* and A-substituted variants after 6 hours of DMSO control or DEX induction. Numbers denote sample sizes per group. p-values derived from one-tailed Wilcoxon rank-sum test (*pPLT2*) or t-tests (others).

Table S1. Genes per PLT clade with PLAZA5 gene name and corresponding identifier used in this study.

Table S2. Motif position-specific scoring matrices found per PLT clade and dataset with corresponding identifier.

Table S3. Significant FIMO hits of all motifs on PLT upstream sequences.

Table S4. BOX occupancy and position across *PLT1/2* orthologs.

Table S5. CRISPR mutants generated in this study. Mutation sites are indicated upstream (-) or downstream (+) of the ATG, which is not counted.

Table S6. R software and packages used in this study.

Table S7. Oligonucleotides used in this study**.**
